# Global, regional, and national burdens of congenital heart anomalies from 1990 to 2021, and projections to 2050

**DOI:** 10.3389/fped.2025.1601620

**Published:** 2025-08-18

**Authors:** Erdengqieqieke Ye, Erman Wu, Tong Tang, Xiaolin La

**Affiliations:** ^1^ Department of Prenatal Diagnosis, Reproductive Medicine Center, The First Affiliated Hospital of Xinjiang Medical University, Urumqi, Xinjiang, China; ^2^ Department of Neurosurgery, The First Affiliated Hospital of Xinjiang Medical University, Urumqi, Xinjiang, China; ^3^ Department of Computer Science and Information Technologies, Elviña Campus, University of A Coruña, A Coruña, Spain; ^4^ Center for Reproductive Medicine, First Affiliated Hospital of Xinjiang Medical University, Urumqi, China

**Keywords:** congenital heart anomalies, global burden of disease, socio-demographic index, sex-stratified analysis, disability-adjusted life years, years lived with disability

## Abstract

**Background:**

Congenital heart anomalies (CHAs) are the most prevalent birth defects, significantly impacting pediatric populations and healthcare systems worldwide. This study provides a comprehensive analysis of the global burden of CHAs, focusing on sex-stratified trends across Socio-Demographic Index (SDI) quintiles from 1990 to 2021.

**Methods:**

Utilizing data from the Global Burden of Disease (GBD) study, we assessed sex-disaggregated metrics including prevalence, incidence, mortality, disability-adjusted life years (DALYs), years of life lost (YLLs), and years lived with disability (YLDs) for CHAs in 21 GBD regions and 204 countries.

**Results:**

CHA prevalence and incidence remained stable, with higher incidence in lower SDI regions. Mortality rates, age-standardized DALYs, and YLLs consistently decreased, with males showing higher rates, particularly in low SDI areas. YLDs increased in non-high SDI regions, indicating a growing disability burden. Globally, age-standardized prevalence rates showed a downward trend in countries like China and Ethiopia, while an upward trend was seen in Turkmenistan and Argentina. Incidence rates generally decreased, except in Spain and France. Age-standardized death rates nearly universally declined. YLDs increased in over half of the nations, including Belgium and Spain. Correlation analysis revealed increasing trends of prevalence and YLDs with rising SDI, while incidence, deaths, DALYs, and YLLs demonstrated a declining trend with SDI elevation. From 2022 to 2050, the prevalence of congenital heart anomalies is projected to rise significantly, especially in the 0−4 age group, while mortality rates are expected to continue a slow downward trend, particularly in the 20−69 age group.

**Conclusions:**

The burden of CHAs is influenced by sex and SDI, with a projected increase in prevalence and a continued decline in mortality. The increasing trend of YLDs highlights the need for targeted public health strategies to address the growing disability burden and ensure global health equity.

## Introduction

Globally, congenital heart anomalies (CHA) represent a significant burden of disease, contributing to substantial morbidity and mortality, particularly among pediatric populations (1–3). In 2019, there were 8.52 million cases and 0.55 million fatalities attributed to congenital birth defects, with CHAs constituting the predominant group globally (4). The impact of CHA extends beyond individual health, affecting families, healthcare systems, and society at large (5–7). The burden of CHA is not uniformly distributed, it exhibits sex disparities, age variations, and significant regional and national differences (8). Understanding the uneven distribution of CHA across different Socio-demographic Index (SDI) regions, sex, and age groups is crucial for developing targeted interventions to address these disparities (9).

CHAs are the most common type of birth defect, and their management is a critical component of global child health initiatives (10). International policies, such as the Sustainable Development Goals (SDG) set by the World Health Organization (WHO), aim to reduce neonatal and under-five mortality rates (11), which are directly influenced by the prevalence and outcomes of CHA. Efforts to improve prenatal care, screening, and access to pediatric cardiac care are essential in mitigating the disease burden (12, 13). The prevention of congenital birth defects necessitates a multifaceted approach that encompasses prenatal screening, nutritional support, vaccination programs, and the avoidance of detrimental exposures (14). Prenatal screening, which includes ultrasound and blood testing, is instrumental in identifying potential fetal abnormalities early in gestation, allowing for early decision-making and possible corrective measures. Low-resource settings often lack access to advanced diagnostics (e.g., fetal echocardiography, genetic screening), leading to underreporting of mild/asymptomatic cases and delayed interventions. In high-SDI regions, improved diagnostics increase detected prevalence but reduce mortality through early management (15).

Nutritional supplementation is also paramount, with a particular emphasis on folic acid and iodine (16). The study suggests that maternal folic acid supplementation before pregnancy is associated with a reduced risk of congenital heart defects, and lower dietary folate intake during pregnancy is linked to an increased risk of CHDs (17). Preconception folic acid supplementation and adequate dietary folate intake are crucial for the prevention of congenital heart defects (18).

Pregnant women should abstain from alcohol, tobacco, and illicit drugs, as these substances can elevate the risk of birth defects (19). Maintaining a healthy lifestyle, replete with a balanced diet and regular physical activity, is supportive of maternal health and avoiding CHA (20).

The significance of our study lies in its ability to provide insights into the changing landscape of CHA and its impact on global health. Our findings reveal that incidence and years lived with disability (YLDs) increase with higher SDI, indicating a complex relationship between economic development and health outcomes. Additionally, the higher disability-adjusted life years (DALYs) and years of life lost (YLLs) among males compared to females underscore the need for sex-specific health policies. By examining these trends, we can better allocate resources and develop strategies to reduce the global burden of CHA.

The study builds on this foundation by examining long-term trends and the impact of policy changes on the incidence and mortality of CHA. Additionally, the study provides insights into the potential future trends of disease burdens attributable to CHA, emphasizing the need for continued vigilance and intervention strategies to mitigate the impact of these conditions. Through this comprehensive analysis, we aim to contribute to the body of knowledge on CHA and inform future policy and research directions.

## Methods

### Data source

The Global Burden of Disease (GBD) 2021 study quantified the impact of 371 diseases and injuries across 204 nations from 1990 to 2021, encompassing metrics such as prevalence, incidence, mortality, and DALYs, including YLDs and YLLs (21). For CHA, the study utilized ICD−11 codes BE14, LA8Z, PK80.10–13, and corresponding ICD−10 codes Q20.8–21.9, 22.3, 24.5, 24.8, and 24.9. The Socio-demographic Index (SDI), a key development indicator linked to health outcomes, was also utilized. Data for this research are accessible via the Institute for Health Metrics and Evaluation (IHME) (https://www.healthdata.org/) and (https://www.healthdata.org/data-tools-practices/data-sources).

In studies on Years Lived with Disability (YLD) and Disability - Adjusted Life Years (DALYs) for congenital heart disease (CHD), “disability” covers all health - related functional limitations, symptoms, and impacts on quality of life from birth to adulthood. It includes: (1) pre - treatment disability like cyanosis, exercise intolerance, poor growth, and developmental delays in untreated CHD. (2) Residual disability after surgery or catheter interventions, such as cardiac dysfunction, activity limits, and medication side - effects. (3) Treatment - related disability from post - surgical complications, arrhythmias, prosthetic device issues, and surgical sequelae. (4) Long - term disability from chronic problems like heart failure, sudden cardiac death risk, neurodevelopmental issues, and psychosocial impacts.

Disability is measured by using severity - stratified disability weights (from 0.033 for mild asymptomatic CHD to 0.324 for severe symptomatic cases) throughout the patient's life, regardless of treatment. This method assesses the overall health impact of CHD, without differentiating between “natural” and “treatment - related” disability (22–24).

### Statistical analysis

The Estimated Annual Percentage Change (EAPC) is a key metric for assessing trends in Age-Standardized Rates (ASR) over time. Within a regression model formatted as (y = α + β x  + ε), y represents the annual rate change per 100,000 people, α is the intercept, x denotes the calendar year, and *ε s*ignifies the error term. The EAPC is calculated using the formula 100 × [exp(β) - 1], with its 95% confidence interval (CI) derived from the linear regression model parameters. Statistical significance was set at a two-sided *p*-value of less than 0.05. These analyses were performed using R software, version 4.4.1. Concurrently, we employed the Pearson correlation coefficient to assess the correlation between ASR and the SDI, with statistical significance defined by a *p*-value of less than 0.001.

### Projection analysis

The Bayesian Age-Period-Cohort (BAPC) R package, a statistical tool for projecting future disease burden within a Bayesian framework, was not applied to GBD 2021 due to the availability of less than five years of incidence data. Utilizing age-specific population data from 1990 to 2021 and projected figures for 2022 to 2050, we analyzed trends in prevalence and mortality across various age groups. These data were also used to forecast future prevalence, deaths, DALYs, YLDs, and YLLs for congenital anomalies from 2022 to 2050. The BAPC package's default settings were employed for this analysis.

## Results

### Sex-stratified analysis of disease burden in global and SDI quintile from 1990 to 2021

**Figure 1:**
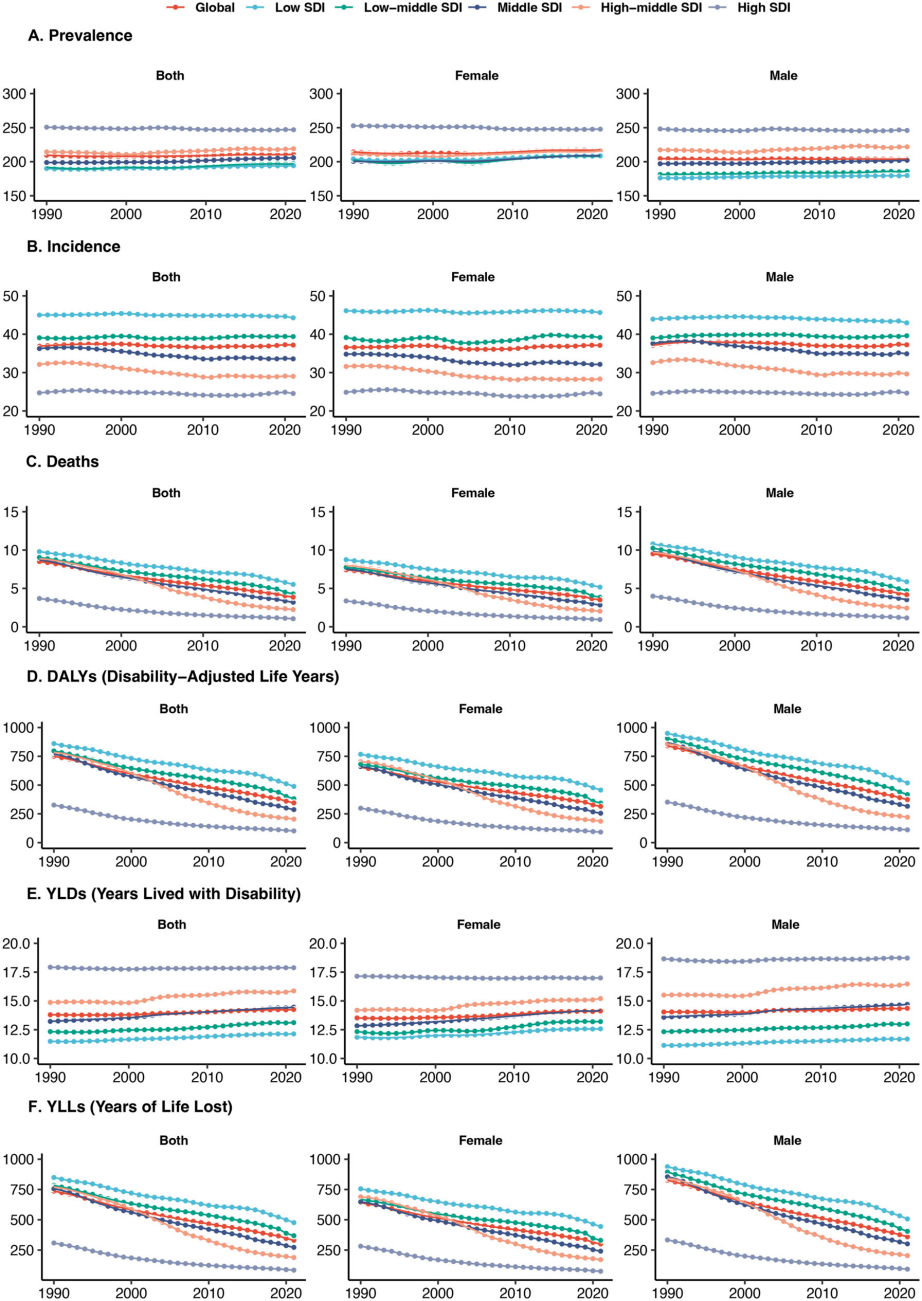
Sex-disaggregated global disease burden rates of congenital heart anomalies by SDI quintile from 1990 to 2021. **(A)** Prevalence. **(B)** Incidence. **(C)** Deaths. **(D)** DALYS. **(E)** YLDs. **(F)** YLLs.

It presents the sex-disaggregated trends and variations in congenital heart anomalies across different SDI quintiles over time. The prevalence and incidence of congenital heart anomalies, depicted in panels (Figures 1A,B), respectively, have remained relatively stable, with incidence rates being higher in regions with lower SDI. Our results indicate a statistically significant but modest global increase in prevalence, with an overall EAPC of 0.04 (95% CI: 0.03–0.05). This corresponds to an approximate cumulative increase of 1.24% over the 31-year period. Notably, the upward trend was more pronounced in females (EAPC: 0.08, 95% CI: 0.06–0.10) compared to males (EAPC: 0, 95% CI: −0.01–0.01). In contrast, there has been a consistent decrease in mortality rates, age-standardized DALYs (Figures 1C,D), and YLLs, with males consistently showing higher rates, particularly in low SDI areas. Notably, age-standardized YLDs have shown an increasing trend in non-high SDI regions, indicating a growing burden of disability (Figures 1E,F). Concurrently, age-standardized YLLs are disproportionately higher in males.

There is no significant sex-based difference in the number of people with congenital heart disease prevalence and incidence across GBD regions (Supplementary Figures S1A,B). However, the number of deaths and DALYs are significantly higher among males, indicating a substantial burden of mortality (Supplementary Figures S1C,D). YLDs exhibit an increasing trend across all SDI regions, suggesting a rising burden of disability. In contrast, YLLs are substantially higher in males compared to females (Supplementary Figures S1E,F).

### Sex-stratified disease burden in 21 distinct GBD regions in 1990 and 2021

**Figure 2:**
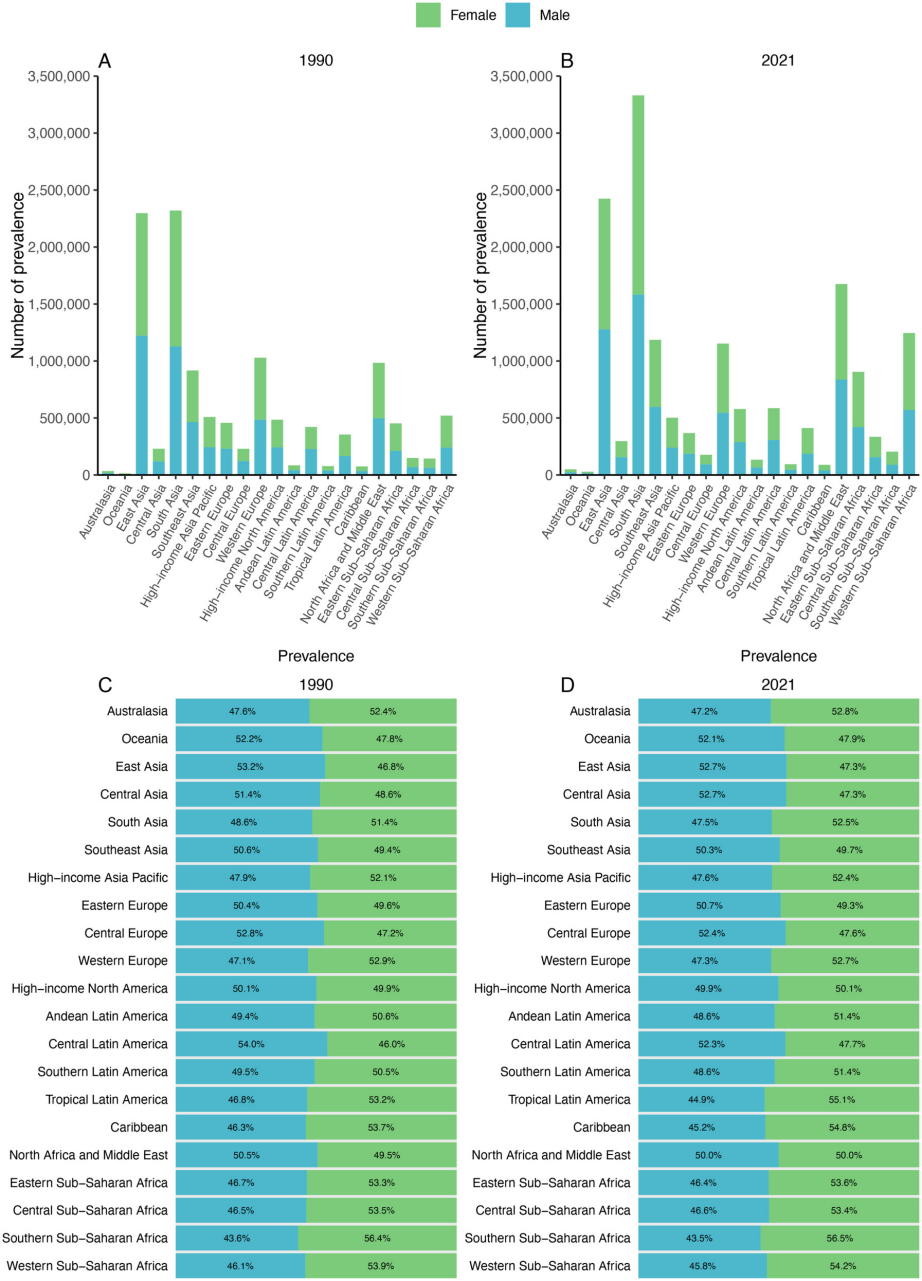
Contribution of prevalence cases by the 21 GBD regions to congenital heart anomalies numbers and proportions, for the years 1990 **(A)** and 2021 **(B)** Additionally, the proportion of prevalence cases attributed to each congenital heart anomalies is depicted for 1990 **(C)** and 2021 **(D)**.

The sex-specific number of prevalence and prevalence percentages across 21 GBD regions for the years 1990 and 2021 are presented in (Figures 2A,B). The amount of prevalence in 1990, with South Asia exhibiting the highest counts at approximately 1,191,604 for females and 1,128,855 for males. By 2021, these numbers have escalated to 1,747,665 for females and 1,583,144 for males in South Asia, marking the most substantial increase in the number of prevalence (Table 1).

**Table 1:** EAPC of age-standardized prevalence rates of congenital heart anomalies and the temporal trends from 1990 to 2021.

Characteristics	1990		2021		1990–2021
	Number, prevalence no. ×10^3^ (95% UI)	Age-standardized prevalence rate per 100,000 (95% UI)	Number, prevalence no. ×10^3^ (95% UI)	Age-standardized prevalence rate per 100,000 (95% UI)	EAPC of Age-standardized prevalence rate (95% CI)
Global	11,787.06 (10,500.37–13,054.73)	209.53 (186.4–231.49)	15,774.46 (14,041.23–17,389.52)	210.7 (187.92–232.48)	0.04 (0.03–0.05)
Female	5,918.04 (5,277.16–6,546.64)	214.38 (190.76–236.98)	8,054.55 (7,147.69–8,900.74)	217.2 (193.26–240.16)	0.08 (0.06–0.1)
Male	5,869.02 (5,215.77–6,500.34)	204.52 (181.66–226.41)	7,719.91 (6,861.11–8,515.57)	204.17 (181.75–225.77)	0 (−0.01–0.01)
Low SDI	1,249.69 (1,096.27–1,405.22)	190 (168.27–212.22)	2,577 (2,271.17–2,865.3)	194.17 (171.51–216.06)	0.09 (0.09–0.1)
Low-middle SDI	2,642.29 (2,333.36–2,939.25)	191.75 (169.02–213.02)	3,822.62 (3,396.45–4,241.02)	196.9 (174.96–218.65)	0.13 (0.11–0.15)
Middle SDI	3,666.15 (3,247.04–4,059.14)	198.86 (176.11–220.09)	4,618.73 (4,108.76–5,098.99)	205.81 (183.42–226.82)	0.12 (0.11–0.14)
High-middle SDI	2,190.05 (1,951.29–2,421.62)	214.42 (191.08–238.22)	2,424.93 (2,151.64–2,675.78)	219.08 (195.74–242.45)	0.11 (0.08–0.14)
High SDI	2,028.72 (1,833.32–2,218.14)	250.69 (226.85–272.64)	2,319.27 (2,079.34–2,546.35)	246.92 (222.72–271.28)	−0.05 (−0.06–0.04)
East Asia	2,297.42 (2,016.4–2,547.97)	189.43 (166.35–210.34)	2,424.3 (2,134.9–2,699.2)	195.8 (173.55–216.6)	0.15 (0.13–0.17)
Southeast Asia	917.08 (805.69–1,019.27)	177.6 (156.19–197.24)	1,185.58 (1,041.84–1,313.77)	179.93 (158.29–199.55)	0.03 (0.02–0.04)
Oceania	13.91 (12.23–15.65)	176.29 (155.51–197.29)	28.38 (24.74–31.79)	177.71 (155.25–199.25)	0.01 (0–0.02)
Central Asia	230.08 (201.96–258.4)	294.09 (258.24–329.31)	296.86 (262.77–330.69)	306 (271.06–340.42)	0.06 (0.03–0.1)
Central Europe	229.79 (205.93–254.28)	209.15 (187.38–232.28)	177.44 (158.31–195.29)	213.81 (191.78–238.39)	0.06 (0.03–0.08)
Eastern Europe	457.25 (408.2–505.7)	225.7 (201.42–250.65)	367.76 (328.36–406.84)	225.83 (202.08–251.2)	−0.02 (−0.03–0)
High-income Asia Pacific	508.8 (451.04–564.85)	317.65 (284.45–351.28)	502.67 (442.57–560.25)	312.02 (280.79–343.6)	−0.02 (−0.07–0.03)
Australasia	34.36 (30.74–37.86)	184.93 (165.37–204.46)	49.54 (44.33–54.9)	188.81 (168.82–209.44)	0.01 (−0.04–0.06)
Western Europe	1,029.07 (930.29–1,125.67)	293.27 (266.6–318.48)	1,152.52 (1,025.3–1,272.73)	298.44 (269.05–326.88)	0.07 (0.05–0.08)
Southern Latin America	77.76 (69.07–85.82)	154.22 (137.08–170.19)	94.22 (85.07–103.62)	163.27 (147.61–180.11)	0.13 (0.11–0.16)
High-income North America	484.95 (432.46–537.71)	190.11 (169.97–211.34)	578.5 (522.49–634.28)	189.71 (171.02–210.01)	−0.09 (−0.18–0)
Caribbean	74.81 (66.93–82.65)	201.85 (180.58–222.77)	89.03 (79.67–98.63)	197.77 (177.33–218.64)	−0.09 (−0.1–0.07)
Andean Latin America	84.35 (75.87–93.07)	198.93 (178.84–218.61)	133.5 (120.89–145.62)	204.3 (184.87–222.81)	0.08 (0.04–0.11)
Central Latin America	421.41 (378.96–463.33)	230.64 (206.85–253.79)	586.01 (527.31–643.59)	242.43 (218.57–266.16)	0.13 (0.1–0.17)
Tropical Latin America	354.77 (319.53–390.13)	222.17 (199.77–244.37)	411.51 (370.94–450.24)	194.9 (176.19–213.75)	−0.49 (−0.57–0.41)
North Africa and Middle East	984.12 (881.02–1,088.4)	259.1 (230.73–287.41)	1,675.71 (1,491.59–1,850.48)	269.42 (239.68–297.58)	0.15 (0.13–0.18)
South Asia	2,320.46 (2,040.14–2,595.54)	179.43 (159.06–199.59)	3,330.81 (2,964.17–3,697.81)	187.82 (167.12–209.07)	0.24 (0.2–0.27)
Central Sub-Saharan Africa	149.3 (131.21–169.78)	204.54 (181.03–230.38)	334.86 (292.71–376.08)	204.18 (179.19–228.5)	0 (−0.01–0)
Eastern Sub-Saharan Africa	452.49 (398.57–505.65)	179.54 (157.82–199.64)	904.59 (797.51–1,010.85)	179.61 (157.97–199.84)	0 (0–0.01)
Southern Sub-Saharan Africa	143.98 (128.14–162.33)	248.57 (219.52–280.31)	205.01 (181.44–229.91)	254.11 (225.18–285.14)	0.08 (0.06–0.09)
Western Sub-Saharan Africa	520.88 (457.59–585.89)	206.51 (182.25–231.24)	1,245.64 (1,101.72–1,391.23)	207.94 (183.93–231.93)	0.02 (0.02–0.03)

South Asia exhibited the highest increasing trend with an EAPC of 0.24 (95% CI: 0.20–0.27), while some regions showed stable or declining trends—for example, certain low-SDI regions had negative EAPCs as low as −0.49 (95% CI: −0.57 to −0.41).

Shifts in sex ratio are most notable in Tropical Latin America, where it shows a 1990 prevalence percentage of 53.2% for females and 46.8% for males. By 2021, this ratio has increased to 55.1% for females and 44.9% for males. In most GBD regions, females are overrepresented compared with males in prevalence.

The most significant increase in incidence rates was observed in Western Sub-Saharan Africa. In Andean Latin America, the incidence rate among men notably surpassed that of women, rising from 60.6% in 1990 to 61.8% in 2021 (Supplementary Figure S2 and Table 2). A decline in mortality was recorded across all 21 GBD regions, with particularly pronounced reductions noted in East Asia, South Asia, and North Africa and the Middle East. Consistently across GBD regions, male mortality rates exceeded those of females (Supplementary Figure S3 and Table 3). DALYs and YLLs exhibited a downward trend in the majority of GBD regions; however, an increase was observed in Western Sub-Saharan Africa. As of 2021, the burden of DALYs and YLLs remained greater for men than for women across all 21 GBD regions (Supplementary Figures S4, S6). YLD increased across all 21 distinct GBD, with South Asia experiencing the most pronounced rise. With exceptions for Sub-Saharan Africa, the Caribbean, Tropical Latin America, and High-Income Asia Pacific regions where this pattern did not hold true, the proportion of YLDs among men consistently outstripped that among women (Supplementary Figure S5 and Supplementary Tables S1–S3).

**Table 2:** EAPC of age-standardized incidence rates of congenital heart anomalies and the temporal trends from 1990 to 2021.

Characteristics	1990		2021		1990–2021
	Number, incidence no. ×10^3^ (95% UI)	Age-standardized incidence rate per 100,000 (95% UI)	Number, incidence no. ×10^3^ (95% UI)	Age-standardized incidence rate per 100,000 (95% UI)	EAPC of Age-standardized incidence rate (95% CI)
Global	2,362.97 (1,862.26–3,064.41)	36.88 (29.06–47.82)	2,300.33 (1,813.14–2,967.23)	37.18 (29.31–47.96)	−0.04 (−0.06–0.01)
Female	1,129.79 (895.21–1,457.7)	36.59 (28.99–47.21)	1,108.34 (878.45–1,428.15)	37.11 (29.41–47.81)	0.01 (−0.02–0.05)
Male	1,233.18 (970.06–1,609.25)	37.14 (29.22–48.47)	1,191.98 (937.42–1,546.98)	37.26 (29.3–48.35)	−0.09 (−0.12–0.06)
Low SDI	477.91 (376.68–623.78)	45 (35.47–58.74)	765 (602.68–990.46)	44.28 (34.88–57.33)	−0.04 (−0.05–0.03)
Low-middle SDI	726.27 (573.85–941.78)	39.08 (30.88–50.67)	734.83 (581.39–956.07)	39.38 (31.16–51.24)	0.03 (0.01–0.05)
Middle SDI	726.52 (567.45–952.07)	36.26 (28.32–47.52)	514.06 (395.38–679.96)	33.59 (25.83–44.43)	−0.33 (−0.37–0.29)
High-middle SDI	281.97 (219.77–367.42)	32.12 (25.04–41.86)	163.18 (128.95–211.23)	29.02 (22.93–37.56)	−0.46 (−0.52–0.4)
High SDI	148.66 (125.13–180.12)	24.73 (20.82–29.97)	121.56 (101.07–148.8)	24.56 (20.42–30.07)	−0.13 (−0.17–0.08)
East Asia	452.16 (348.09–599.75)	39.42 (30.35–52.29)	179.63 (136.69–242.92)	32.56 (24.78–44.03)	−0.92 (−1.01–0.82)
Southeast Asia	209.33 (163.44–272.85)	35.32 (27.58–46.04)	182.74 (141.5–238.88)	33.89 (26.24–44.3)	−0.17 (−0.19–0.15)
Oceania	4.09 (3.23–5.31)	37.93 (29.97–49.24)	8.1 (6.33–10.47)	39.43 (30.84–51.01)	0.14 (0.12–0.16)
Central Asia	45.19 (36.15–56.33)	47.88 (38.31–59.69)	49.46 (39.87–61.68)	50.36 (40.6–62.81)	0.02 (−0.01–0.05)
Central Europe	22.96 (18.71–28.6)	27.82 (22.67–34.65)	14.16 (11.59–17.73)	28.15 (23.04–35.24)	−0.13 (−0.2–0.06)
Eastern Europe	44.18 (34.67–56.92)	30.78 (24.15–39.65)	27.18 (21.65–34.74)	31.52 (25.11–40.29)	0.06 (0.04–0.09)
High-income Asia Pacific	28.83 (23.82–35.78)	30.36 (25.09–37.68)	15.94 (13.11–19.79)	27.9 (22.95–34.64)	−0.34 (−0.44–0.24)
Australasia	2.85 (2.29–3.59)	18.69 (15.02–23.54)	3.34 (2.68–4.25)	19.44 (15.58–24.72)	0.16 (0.11–0.2)
Western Europe	48.31 (43.6–54.42)	21.71 (19.59–24.46)	46.5 (39.89–54.98)	23.59 (20.23–27.89)	0.23 (0.22–0.24)
Southern Latin America	12.18 (9.82–15.37)	24.01 (19.36–30.31)	9.25 (7.68–11.43)	24.86 (20.65–30.7)	−0.05 (−0.11–0.01)
High-income North America	54.66 (44.14–69.09)	24.88 (20.1–31.45)	48.84 (39.66–62)	25.06 (20.35–31.8)	−0.09 (−0.17–0.02)
Caribbean	13.04 (10.31–17.63)	30.2 (23.86–40.81)	12.68 (10.3–16.07)	33.24 (27–42.14)	0.32 (0.3–0.33)
Andean Latin America	19.52 (14.91–27.32)	34.68 (26.49–48.53)	20.03 (15.06–28.57)	33.69 (25.33–48.06)	−0.08 (−0.11–0.05)
Central Latin America	76.97 (58.87–104.12)	32.13 (24.57–43.46)	57.08 (43.56–77.65)	30.46 (23.24–41.44)	−0.09 (−0.12–0.06)
Tropical Latin America	55.57 (40.99–78.39)	34.49 (25.44–48.65)	55.83 (41.23–79.85)	33.78 (24.95–48.31)	−0.05 (−0.1–0.01)
North Africa and Middle East	159.79 (132.86–195.49)	30.41 (25.29–37.2)	181 (150.55–223.57)	31.64 (26.31–39.08)	0.09 (0.07–0.1)
South Asia	629.92 (493.66–816.39)	38.21 (29.95–49.52)	597.01 (469.73–777.53)	39.43 (31.03–51.36)	0.11 (0.07–0.15)
Central Sub-Saharan Africa	58.98 (45.67–77.49)	47.86 (37.07–62.89)	96.03 (74.49–126.67)	44.92 (34.85–59.26)	−0.2 (−0.23–0.17)
Eastern Sub-Saharan Africa	187.14 (145.1–248.37)	43.51 (33.74–57.75)	269.69 (208.56–360.22)	41.11 (31.79–54.9)	−0.19 (−0.21–0.18)
Southern Sub-Saharan Africa	30.69 (23.36–42.01)	39.5 (30.07–54.07)	30.19 (22.89–40.92)	38.62 (29.29–52.35)	−0.08 (−0.1–0.07)
Western Sub-Saharan Africa	206.59 (160.76–272.89)	48.18 (37.49–63.63)	395.64 (308.09–519.73)	46.43 (36.16–61)	−0.09 (−0.11–0.08)

**Table 3:** EAPC of age-standardized deaths rates of congenital heart anomalies and the temporal trends from 1990 to 2021.

Characteristics	1990		2021		1990–2021
	Number, deaths no. ×10^3^ (95% UI)	Age-standardized deaths rate per 100,000 (95% UI)	Number, deaths no. ×10^3^ (95% UI)	Age-standardized deaths rate per 100,000 (95% UI)	EAPC of Age-standardized deaths rate (95% CI)
Global	528.95 (310.32–679.09)	8.51 (5.01–10.9)	250.81 (207.82–304.08)	3.86 (3.19–4.7)	−2.38 (−2.44–2.32)
Female	223.14 (124.45–287.29)	7.43 (4.16–9.56)	111.02 (86.93–136.05)	3.51 (2.74–4.29)	−2.22 (−2.29–2.16)
Male	305.81 (164.11–425.81)	9.51 (5.14–13.24)	139.79 (110.88–181.1)	4.18 (3.31–5.42)	−2.5 (−2.56–2.44)
Low SDI	92.47 (34.13–134.9)	9.8 (3.74–14.28)	90.67 (64.96–122.2)	5.53 (4.02–7.44)	−1.61 (−1.7–1.53)
Low-middle SDI	157.72 (84.75–213.75)	9.03 (4.9–12.21)	81.21 (64.19–100.98)	4.29 (3.39–5.34)	−2.06 (−2.18–1.95)
Middle SDI	172.91 (109.84–228.44)	8.72 (5.55–11.52)	55.12 (46.57–66.28)	3.2 (2.68–3.87)	−3.07 (−3.14–3)
High-middle SDI	81.06 (57.65–99.5)	8.87 (6.28–10.91)	16.29 (13.82–18.92)	2.25 (1.87–2.65)	−4.79 (−5–4.58)
High SDI	24.35 (21.24–26.3)	3.69 (3.2–4)	7.26 (6.43–8.46)	1.05 (0.9–1.24)	−3.88 (−4.04–3.73)
East Asia	127.88 (82.12–176.23)	10.98 (7.01–15.14)	20.84 (16.84–25.8)	2.71 (2.15–3.45)	−5.02 (−5.28–4.76)
Southeast Asia	46.65 (23.42–62.79)	8.03 (4.06–10.8)	23.23 (19.11–28.53)	4.09 (3.36–5.04)	−2.21 (−2.32–2.11)
Oceania	1.03 (0.39–1.53)	10.28 (4.03–14.99)	1.75 (0.81–2.62)	9 (4.35–13.32)	−0.35 (−0.48–0.21)
Central Asia	4.24 (3.68–4.79)	4.61 (4.02–5.2)	4.63 (3.67–5.68)	4.68 (3.72–5.74)	0.3 (0–0.6)
Central Europe	5.31 (4.59–5.86)	5.97 (5.14–6.63)	0.92 (0.76–1.06)	1.49 (1.22–1.74)	−4.58 (−4.72–4.44)
Eastern Europe	8.29 (7.43–9.76)	5.2 (4.64–6.18)	2.05 (1.81–2.42)	1.66 (1.41–2.01)	−4.12 (−4.59–3.66)
High-income Asia Pacific	4.3 (3.54–4.79)	3.86 (3.14–4.34)	0.76 (0.62–0.96)	0.79 (0.62–1.04)	−4.89 (−5.03–4.74)
Australasia	0.35 (0.33–0.38)	2.08 (1.93–2.27)	0.18 (0.15–0.22)	0.79 (0.64–0.96)	−3.08 (−3.24–2.91)
Western Europe	8.15 (7.1–8.8)	3.18 (2.75–3.44)	2.43 (2.13–2.84)	0.89 (0.74–1.06)	−4.1 (−4.29–3.91)
Southern Latin America	2.2 (1.85–2.57)	4.34 (3.65–5.07)	1.04 (0.88–1.25)	2.47 (2.04–3)	−1.64 (−1.85–1.43)
High-income North America	6.65 (5.83–7.18)	2.81 (2.45–3.05)	2.88 (2.62–3.53)	1.1 (0.97–1.35)	−2.77 (−2.9–2.63)
Caribbean	4.11 (3.1–5.09)	9.84 (7.42–12.17)	2.63 (1.65–4.49)	6.72 (4.2–11.34)	−0.97 (−1.17–0.78)
Andean Latin America	5.29 (2.89–6.87)	9.87 (5.42–12.77)	2.47 (1.88–3.17)	4.05 (3.07–5.19)	−2.29 (−2.45–2.13)
Central Latin America	12.27 (10.83–13.92)	5.37 (4.76–6.07)	8.83 (6.99–11.13)	4.39 (3.43–5.54)	−0.41 (−0.59–0.23)
Tropical Latin America	8.53 (7.31–9.83)	5.26 (4.51–6.06)	5.31 (4.36–6.38)	3.04 (2.47–3.68)	−1.13 (−1.47–0.8)
North Africa and Middle East	95.84 (42.9–135.85)	18.75 (8.45–26.55)	35.27 (28.07–43.67)	5.99 (4.76–7.42)	−3.46 (−3.55–3.37)
South Asia	115.43 (72.64–154.22)	7.41 (4.67–9.87)	57.26 (41.72–78.5)	3.63 (2.62–5.02)	−1.93 (−2.06–1.81)
Central Sub-Saharan Africa	7.8 (2.44–14.24)	7.1 (2.34–12.78)	6.54 (4.11–10.62)	3.24 (2.03–5.27)	−2.22 (−2.43–2.01)
Eastern Sub-Saharan Africa	28.86 (8.25–55.99)	7.55 (2.29–14.74)	23.55 (14.99–41.89)	3.74 (2.44–6.63)	−1.99 (−2.1–1.89)
Southern Sub-Saharan Africa	1.94 (1.58–2.51)	2.68 (2.17–3.44)	1.71 (1.16–2.27)	2.16 (1.46–2.86)	−0.4 (−0.5–0.3)
Western Sub-Saharan Africa	33.82 (9.34–51.42)	9.22 (2.74–13.91)	46.52 (28.52–65.07)	5.96 (3.8–8.25)	−1.07 (−1.19–0.96)

### Global trends in the burden of congenital heart anomalies across 204 countries and territories

**Figure 3:**
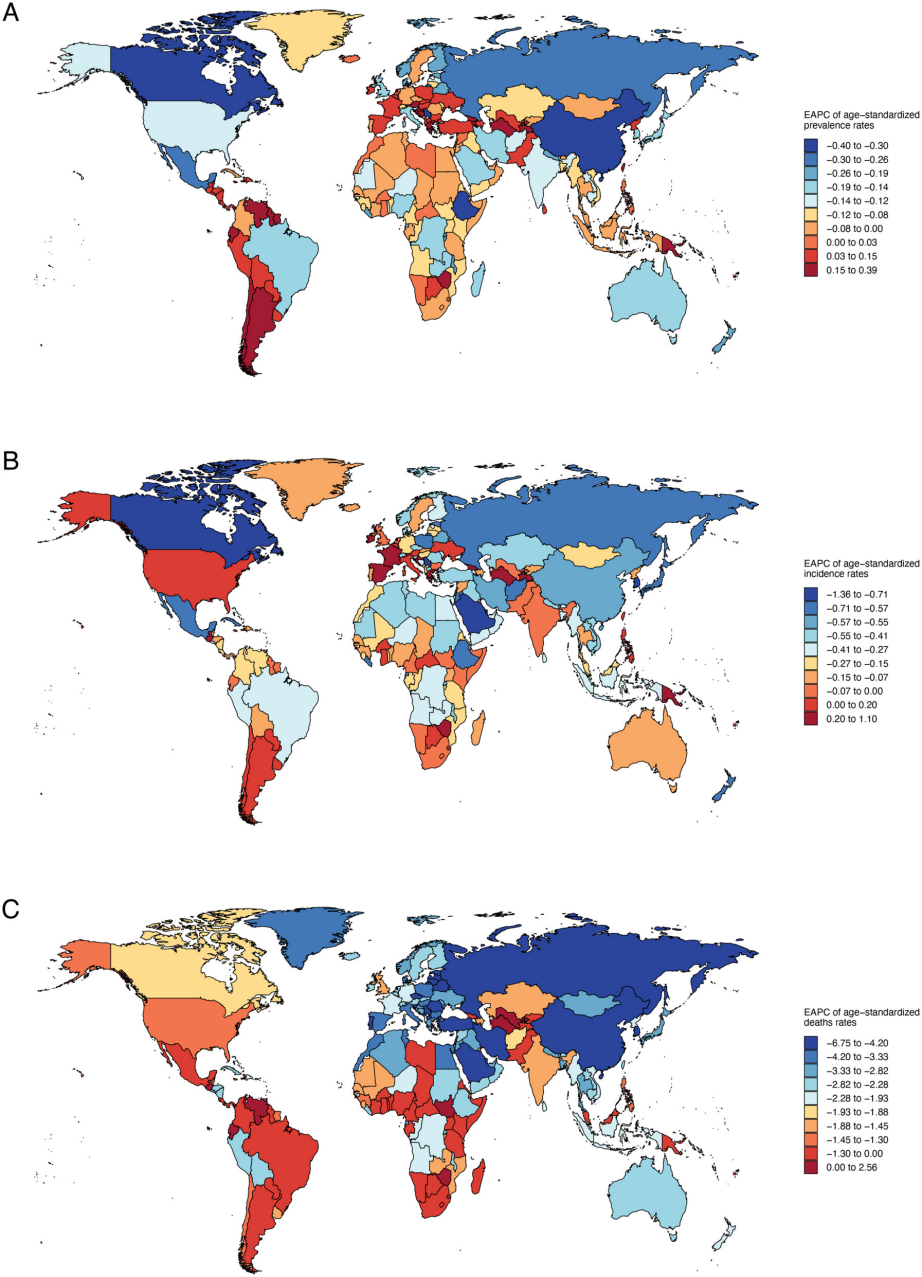
Annual percentage change in national age-standardized prevalence, incidence, death rates of congenital heart anomalies in 2021. **(A)** Prevalence. **(B)** Incidence. **(C)** Deaths.

The EAPC data (Figure 3) reveals significant variations in health metrics across countries. For Age-Standardized Prevalence Rates (ASPR), a notable downward trend is observed in China, Ethiopia, and Canada, indicating a reduction in disease prevalence. In contrast, an upward trend is seen in Turkmenistan, Argentina, and Chile, suggesting an increase in disease burden in these regions (Supplementary Table S4). Incidence Rates (ASIR) generally show a downward trend, reflecting a global decrease in disease incidence, with the exception of Spain and France, where an upward trend may signal emerging health challenges (Supplementary Table S5). Age-Standardized Death Rates (ASDR) exhibit a nearly universal decline, with the exception of a few countries including the Northern Mariana Islands, American Samoa, Seychelles, and others (Supplementary Table S6).

It shows a general decline in age-standardized DALYs and YLLs, pointing to a reduced disease burden in many countries, yet an upward trend in YLDs affects over half of the nations, including Belgium, Spain, and Austria (Supplementary Table S8). Notably, Turkmenistan, Guatemala, Dominica, and Uzbekistan exhibit rising DALYs and YLLs, suggesting potential health challenges (Supplementary Tables S7, S9, Supplementary Figure S7).

### Age-specific disease burden of congenital heart anomalies in various SDI regions

**Figure 4:**
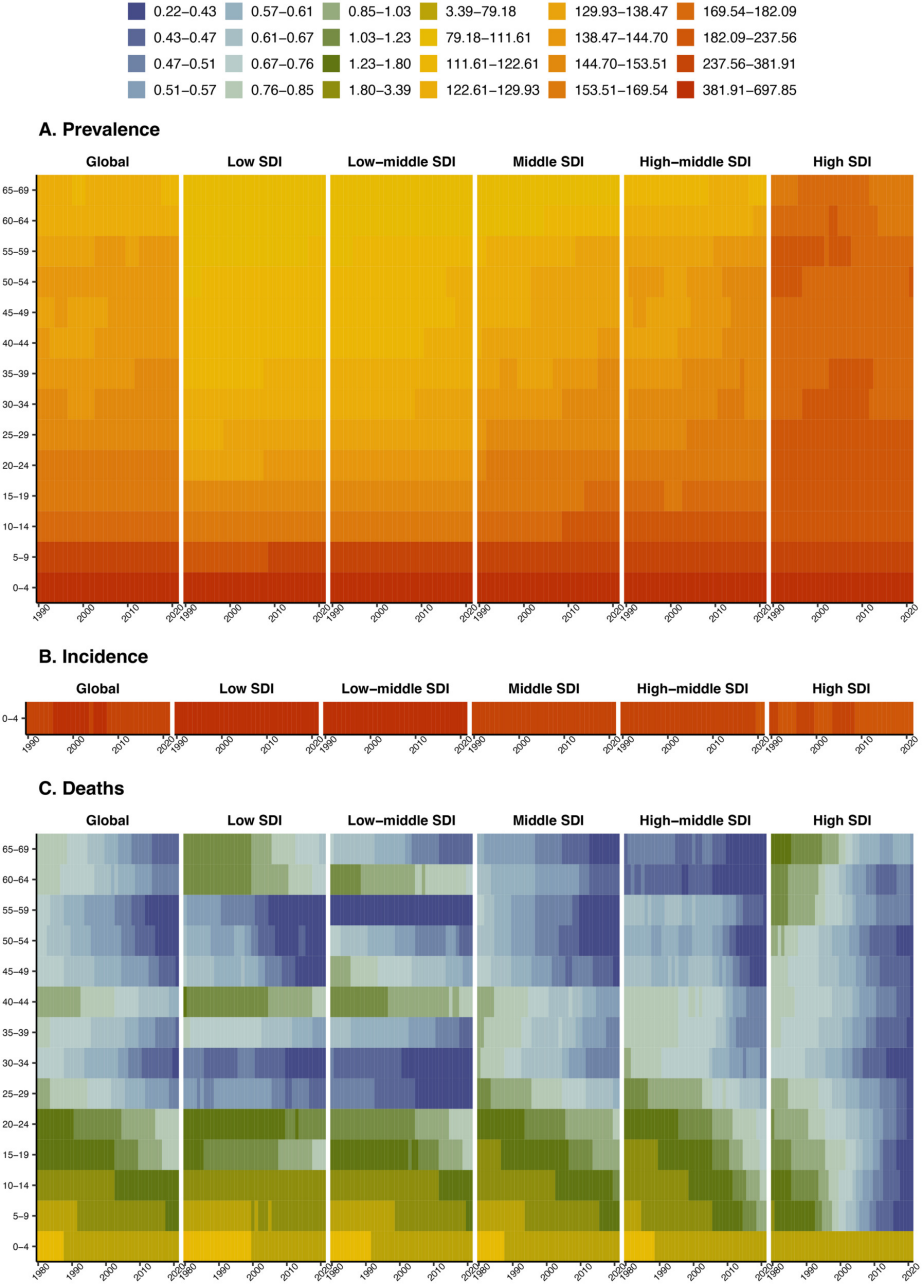
Age-specific disease burden of congenital heart anomalies in various SDI regions from 1990 to 2021. **(A)** Prevalence. **(B)** Incidence. **(C)** Deaths.

It succinctly captures the comparative dynamics of prevalence, incidence, and mortality rates for congenital heart anomalies across age groups and Socio-Demographic Index (SDI) regions from 1990 to 2021. The prevalence rate for children aged 0–4 is notably high in all SDI regions, with a decreasing trend in prevalence as age groups progress from 5 to 9 to 65–69 years. Incidence rates are high in all SDIs, particularly in low and low-middle SDI regions, with a decreasing trend observed in higher SDI regions over the study period. Mortality rates are particularly high in the 0–4 age group across all SDI regions and have shown a decline in recent years. While the 0–24 years age group has relatively high mortality, the 45–59 age group exhibits comparatively lower rates (Figure 4). DALYs and YLLs predominantly burden individuals aged 0–24 years, with a notable decrease in impact across all age groups from 1990 to 2021. YLDs, affecting primarily those aged 0–9 years, exhibit a lower impact in regions with Low SDI, indicating a reduced burden of Congenital Heart Anomalies in these areas (Supplementary Figure S8).

### SDI correlations with congenital heart anomalies burden trends in 21 GBD regions and 204 countries and territories

**Figure 5:**
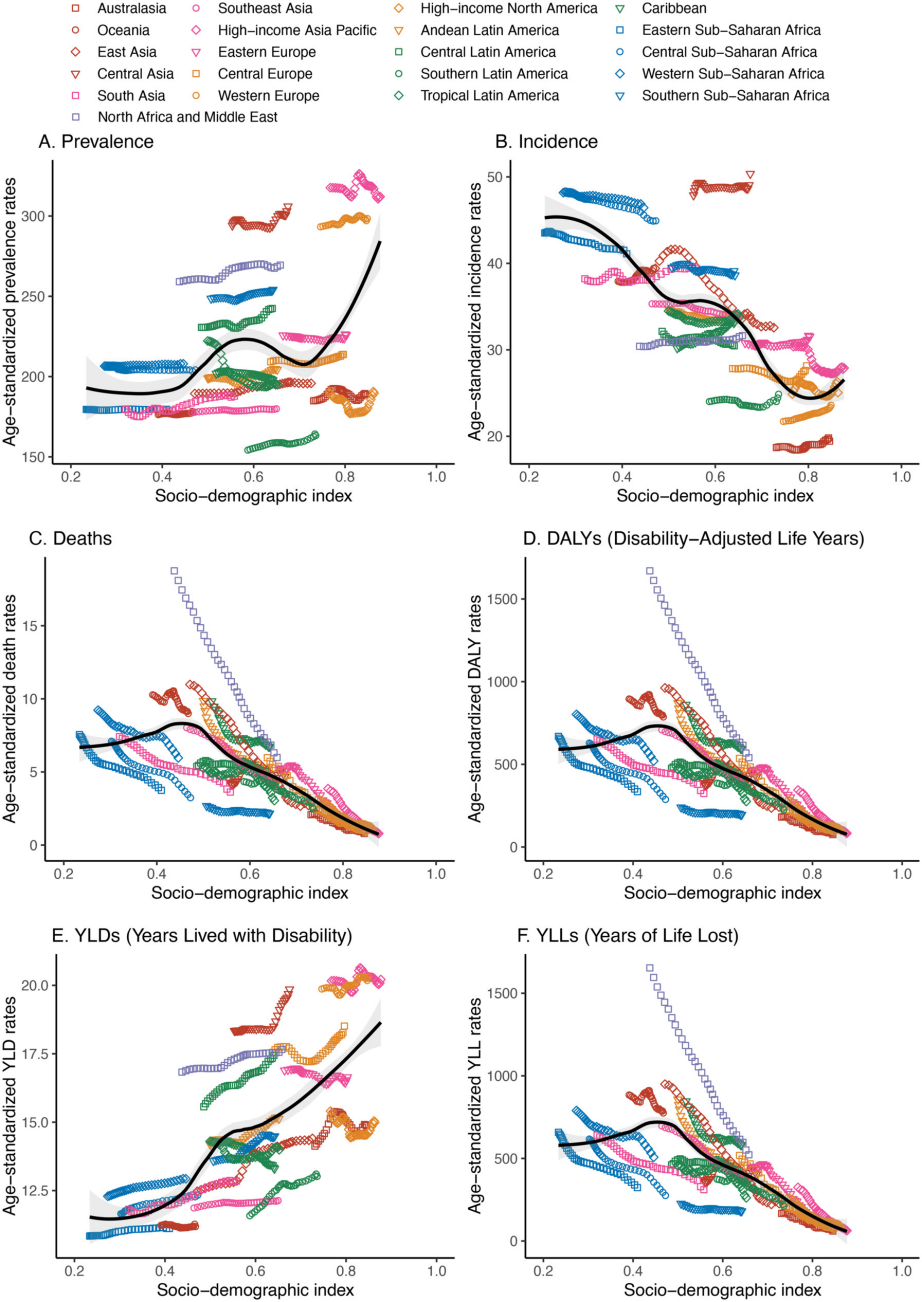
Association between the disease burden of congenital heart anomalies and sociodemographic index across 21 GBD regions from 1990 to 2021. **(A)** Prevalence. **(B)** Incidence. **(C)** Deaths. **(D)** DALYS. **(E)** YLDs. **(F)** YLLs.

Across the 21 GBD regions, both prevalence and YLDs exhibited an increasing trend in conjunction with rising SDI values, with correlation coefficients of 0.3661 and 0.6982, respectively, and a *p*-value below 0.001. Conversely, incidence, deaths, DALYs, and YLLs demonstrated a declining trend with the elevation of SDI, with correlation coefficients of −0.7660, −0.6713, −0.6658, and −0.6707, respectively. Their *p*-values were all less than 0.001 (Figure 5).

**Figure 6:**
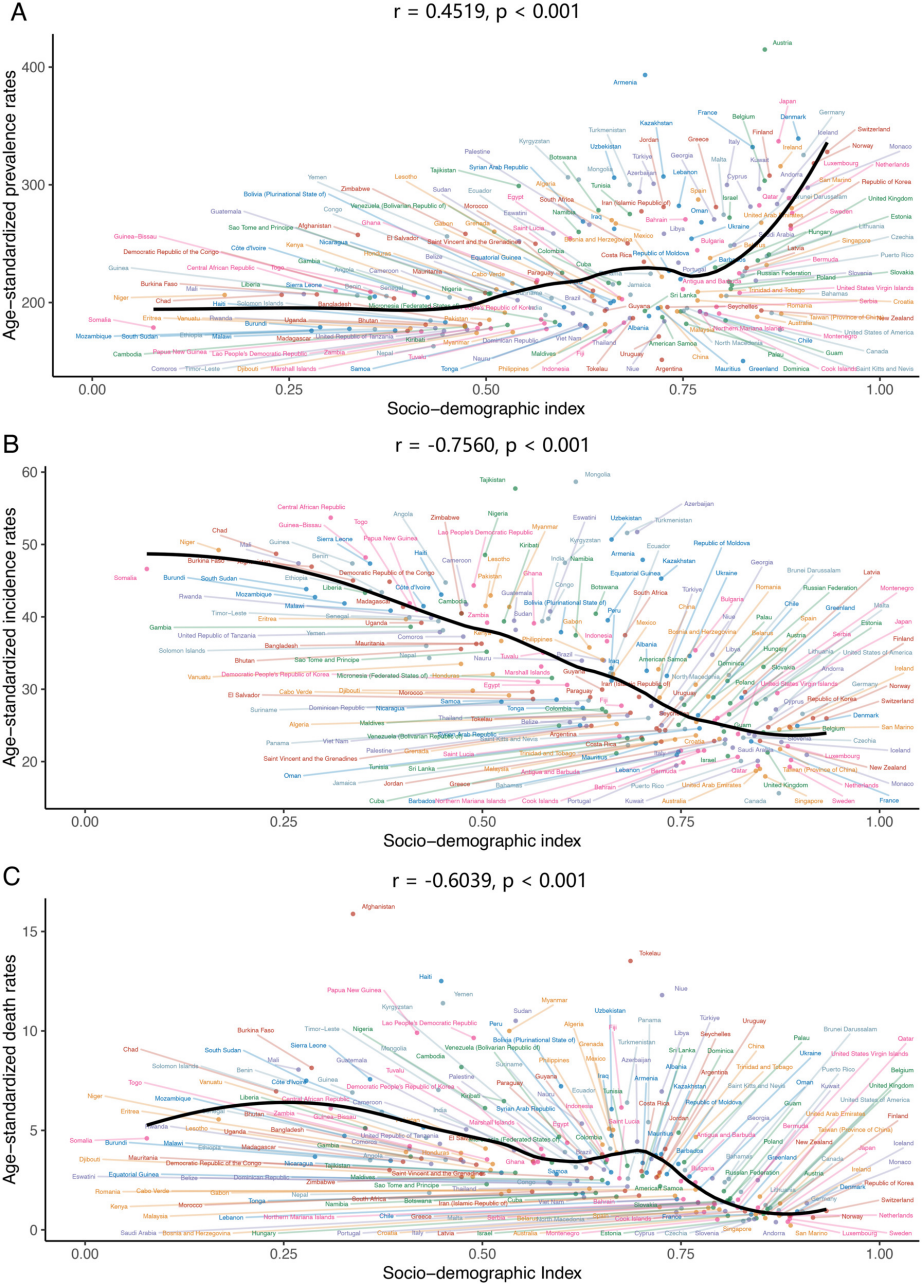
The association between age-standardized prevalence, incidence, deaths rate of congenital heart anomalies and sociodemographic index across 204 countries and territories in 2021. **(A)** Prevalence. **(B)** Incidence. **(C)** Deaths.

In the 204 countries and regions, prevalence and YLDs rose with increasing SDI, with correlation coefficients of 0.4519 and 0.6456, respectively (both *p* < 0.001). The age-standardized incidence rate (ASIR) declined significantly with SDI, with a correlation coefficient of −0.7560 (*p* < 0.001). The age-standardized death rate (ASDR) also decreased with SDI, at −0.6039 (*p* < 0.001) (Figure 6 and Supplementary Figure S9). The trends in age-standardized DALY and YLL rates were closely aligned, though less markedly, with correlation coefficients of −0.5936 and −0.5999, respectively (both *p* < 0.001) (Supplementary Figure S9).

### Projections of congenital heart anomalies from 2022 to 2050

**Figure 7:**
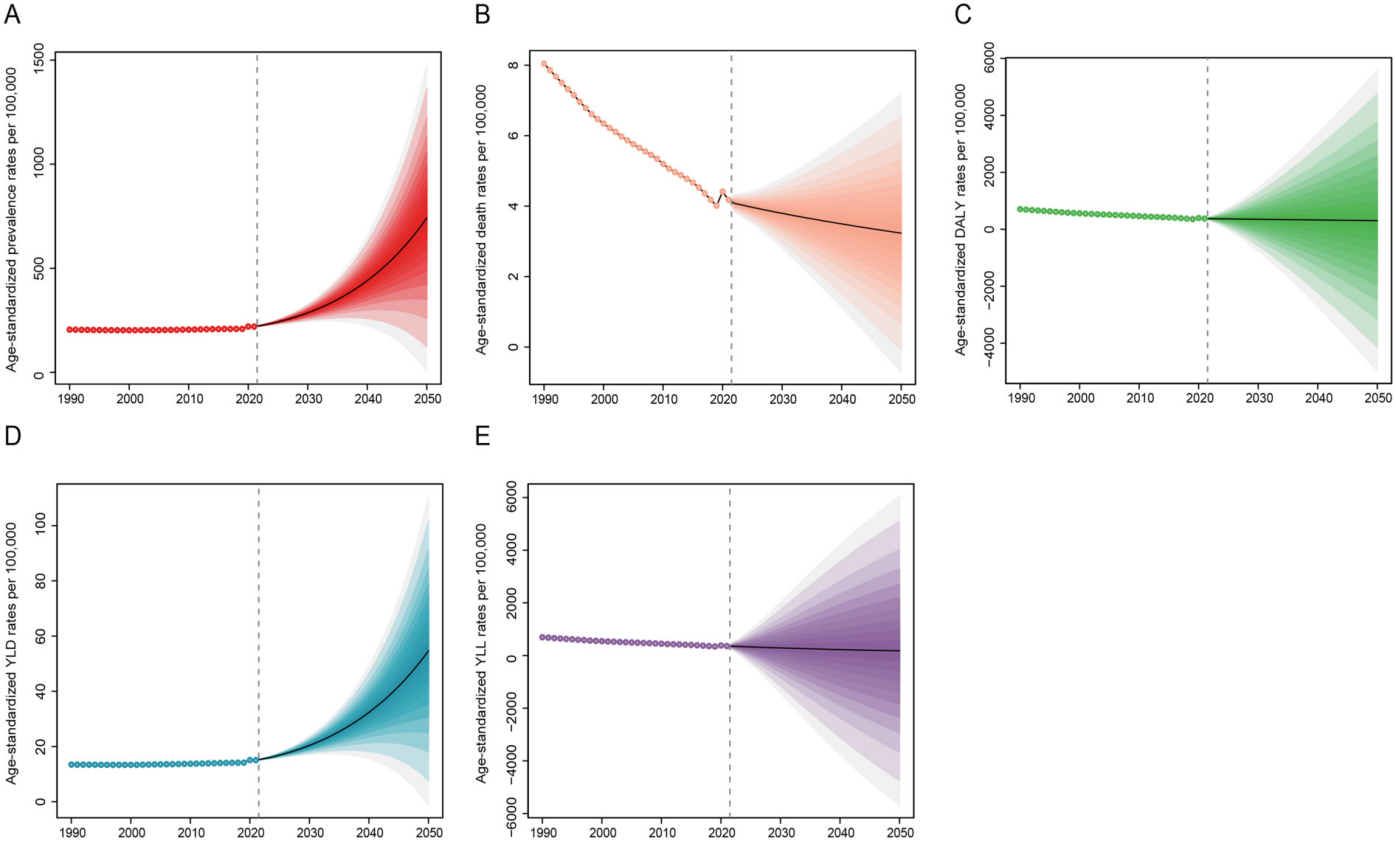
Projection of disease burden of congenital heart anomalies from 2022 to 2050. **(A)** Prevalence. **(B)** Deaths. **(C)** DALYS. **(D)** YLDs. **(E)** YLLs.

From 2022 to 2050, the prevalence of congenital heart anomalies is projected to rise, particularly among the 0–29 age group, with minimal change anticipated in those aged 30 and above (Figure 7 and Supplementary Figure S10). Despite a significant decline in mortality from 1990 to 2021, future rates are expected to continue a slow downward trend, with the 20–69 age group showing a more pronounced reduction (Figure 7 and Supplementary Figure S11). Age-standardized DALY and YLL rates will slightly decrease, while YLD rates will markedly increase (Figure 7). By 2050, the number of prevalence is expected to rise significantly, with the 0–4 age group projected to reach 25,211,160 (95% UI, 6,556,715–43,865,606), a substantial increase from 4,183,259 (95% UI, 4,800,270–3,643,893) in 2021 (Supplementary Table S10). Conversely, the mortality rate for this age group is predicted to drop significantly, from 204,222 (95% UI, 255,409–165,238) to 178,084 (95% UI, 39,675–316,493) (Supplementary Table S11). While DALYs and YLLs are set to increase, YLDs are anticipated to decrease (Supplementary Tables S12–S14).

## Discussion

The GBD 2021 analysis provides critical insights into the evolving burden of congenital heart anomalies. Global trends from 1990 to 2021 show that congenital heart anomalies had stable global prevalence and incidence but declining mortality, DALYs, and YLLs. Males had higher fatality rates, especially in low - SDI regions. YLDs increased significantly in non - high - SDI areas, indicating a shift to a disability - driven burden. Regionally, South Asia had the most cases in 2021 (1.75M females/1.58M males). Western Sub - Saharan Africa had the fastest incidence growth and rising DALYs/YLLs. Sex disparities grew in Latin America. Mortality fell in 97% of 204 countries, but disability (YLDs) worsened in 50%. SDI correlations: Higher SDI quintiles were associated with rising prevalence/YLDs (*r* > 0.64) but falling incidence/mortality (*r* < −0.6). Children aged 0–4 were the most vulnerable across all SDI levels. Projections to 2050 suggest a 500% increase in global cases among 0–4 - year - olds (to 25.2M), with slowly declining mortality and sharply rising YLDs. This shows an epidemiological transition from a lethal to a disabling disease. Low - SDI regions face high incidence, while middle/high - SDI regions deal with increasing disability. Targeted child - focused interventions are needed in poor areas, and disability management in developed economies.

Previous research on CHA has not consistently examined the disease burden from 1990 to 2021 through the lens of sex, age, and SDI (1, 8, 9). Our study addresses this omission, offering a comprehensive overview of CHA's global impact during this period, with detailed analysis across sex, age groups, SDI regions, and countries. It also projects prevalence, mortality, DALYs, YLDs, and YLLs for different age groups from 2022 to 2050, including specific predicted numerical values. This in-depth analysis offers fresh insights into CHA's global public health and informs future health policy and resource allocation.

The findings indicate that CHA prevalence and incidence remained largely stable from 1990 to 2021, with a minor increase. The ASPR rose slightly from 209.53 (186.4–231.49) to 210.7 (187.92–232.48), and the ASIR increased marginally from 36.88 (29.06–47.82) to 37.18 (29.31–47.96). In low SDI areas, age-standardized incidence rates slightly dipped from 45 (35.47–58.74) to 44.28 (34.88–57.33), but remained elevated, indicating a need for targeted interventions to reduce CHA incidence (25, 26). Age-standardized mortality and DALY rates saw a significant drop, from 8.51 (5.01–10.9) to 3.86 (3.19–4.7) and from 750.3 (440.94–960.11) to 345.24 (288.34–422.16), respectively. High SDI areas showed a more pronounced decline in death rates and age-standardized DALY rates, reflecting improved CHA treatment worldwide (5, 27, 28). In 2021, global female CHA prevalence rates were higher at 217.2 (193.26–240.16) compared to males at 204.17 (181.75–225.77), yet female death rates were lower at 3.51 (2.74–4.29) compared to males at 4.18 (3.31–5.42). This could suggest higher susceptibility in male, a protective role for estrogen (29, 30), or superior treatment adherence in female (31). From 1990 to 2021, CHA deaths in South Asia fell significantly, from 115.43 (72.64–154.22) thousand to 57.26 (41.72–78.5) thousand. East Asia is projected to experience the steepest decline in future mortality, with an EAPC of −5.02 (−5.28 to −4.76). The downward death rate trend across all SDI regions points to advancements in CHA treatment, improved public health policies, and better socioeconomic conditions (32, 33). Globally, the disease burden is highest in CHA patients aged 0–4, decreasing with age, except in high SDI regions where advanced medical facilities and treatments have led to improved survival rates, allowing patients to live longer and thus not showing a significant age-related prevalence decline (34). CHA prevalence and YLDs rise with SDI, while incidence, mortality, DALYs, and YLLs fall. Improved diagnostics have led to earlier detection of mild or asymptomatic cases, increasing prevalence (35). The increased survival rate of CHA patients has reduced mortality and DALYs, as many now survive to adulthood or old age.

Looking ahead to 2050, our predictions suggest a rise in the occurrence of CHA, particularly among younger individuals. The death rate is projected to decrease gradually, yet the DALYs are expected to see a substantial increase, maintaining an upward trajectory (Figure 7). While global pediatric populations are declining (UNPD 2022), CHA prevalence is rising disproportionately in low/middle-SDI regions due to: higher fertility rates and improving survival (reducing mortality but increasing lifelong disability burden) (36). With the anticipated growth in the global population, especially in regions with low and medium SDI, an increase in the number of CHA cases is likely, potentially raising prevalence rates. This necessitates targeted interventions. Despite the slow decline in mortality, individuals with CHA may still experience disabilities and a diminished quality of life, resulting in a rise in YLDs. Furthermore, as CHA patients age, they are likely to confront health issues associated with aging, further elevating DALYs (37).

Based on our GBD 2021 analysis, we acknowledge that adult congenital heart disease (ACHD) represents a growing clinical challenge as survival into adulthood has dramatically improved. While the GBD framework classifies congenital heart anomalies as a unified entity without explicit ACHD stratification, our findings likely capture this evolving burden. The sustained high DALYs and YLDs observed across older age groups probably reflect ACHD sequelae—including heart failure, arrhythmias, and complications from previous interventions that characterize this population. Geographic variations in burden may mirror disparities in pediatric cardiac surgical access and lifelong ACHD care infrastructure, which significantly influence outcomes. Recent registry data confirm the ACHD population is expanding and aging, with substantial numbers now surviving beyond 60 years. We agree that future studies should incorporate specific ACHD classifications and age-stratified analyses to better characterize this population's unique burden, as current global health metrics may underestimate the true impact of lifelong complications in ACHD patients who now routinely survive into their sixth and seventh decades.

This study utilizes the most recent GBD 2021 data, providing a more accurate reflection of CHA's current disease burden and enabling more reliable predictions of its future trajectory. We systematically analyzed how sex, age groups, and SDI levels impact CHA across various countries and regions. The EAPC was employed to forecast disease burden trends in different nations, while the BAPC model was used to project CHA disease burden from 2022 to 2050.

There are some limitations in our study: (1) a limitation of this study is its reliance on the BAPC model alone; it did not incorporate ARMIA or Nordped for a broader comparison of future CHA disease burden predictions (38–40). (2) Global diagnostic coverage disparities, (3) birth registration gaps, and (4) interconnected health conditions. As referenced (https://www.OurWorldinData.org), accurate neonatal diagnostics (ultrasound, genomic screening) remain highly inequitable—widely available in high-income countries but critically limited in LMICs, contributing to delayed diagnoses and higher mortality. Significant birth under-registration, particularly in sub-Saharan Africa and South Asia, compromises data accuracy and obscures true disease burden. Additionally, neonatal mortality is intrinsically linked to infections, congenital disorders, and maternal complications, with premature birth being the leading cause of child mortality globally. These diagnostic and registration gaps in LMICs hinder early detection of interconnected risks, preventing timely interventions and perpetuating preventable deaths.

To encapsulate, this study offers a thorough analysis of the global disease burden of CHA and its anticipated trends over the coming decades. The findings underscore the importance of devising health strategies tailored to different SDI regions, particularly those with low SDI, to alleviate the disease burden of CHA. With continued research and intervention, we anticipate a reduction in CHA's impact on global child health and an enhancement in the quality of life for patients in the future.

## Data Availability

The original contributions presented in the study are included in the article/Supplementary Material, further inquiries can be directed to the corresponding author.

## References

[1] GBD 2017 Congenital Heart Disease Collaborators. Global, regional, and national burden of congenital heart disease, 1990–2017: a systematic analysis for the global burden of disease study 2017. Lancet Child Adolesc Health. (2020) 4(3):185–200. 10.1016/S2352-4642(19)30402-X 31978374 PMC7645774

[2] Stallings EB Isenburg JL Aggarwal D Lupo PJ Oster ME Shephard H Prevalence of critical congenital heart defects and selected co-occurring congenital anomalies, 2014–2018: a U.S. Population-based study. Birth Defects Res. (2022) 114:45–56. 10.1002/bdr2.1980 35048540 PMC8915134

[3] Chang CS Hong SY Kim SY Kim YM Sung JH Choi SJ Prevalence of associated extracardiac anomalies in prenatally diagnosed congenital heart diseases. PLoS One. (2021) 16:e0248894. 10.1371/journal.pone.0248894 33735284 PMC7971844

[4] Kang L Cao G Jing W Liu J Liu M. Global, regional, and national incidence and mortality of congenital birth defects from 1990 to 2019. Eur J Pediatr. (2023) 182(4):1781–92. 10.1007/s00431-023-04865-w 36781460

[5] Ma K He Q Dou Z Hou X Li X Zhao J Current treatment outcomes of congenital heart disease and future perspectives. Lancet Child Adolesc Health. (2023) 7:490–501. 10.1016/S2352-4642(23)00076-7 37301213

[6] Mellion K Uzark K Cassedy A Drotar D Wernovsky G Newburger JW Health-related quality of life outcomes in children and adolescents with congenital heart disease. J Pediatr. (2014) 164:781–8. 10.1016/j.jpeds.2013.11.066 24412135

[7] Westhoff-Bleck M Briest J Fraccarollo D Hilfiker-Kleiner D Winter L Maske U Mental disorders in adults with congenital heart disease: unmet needs and impact on quality of life. J Affect Disord. (2016) 204:180–6. 10.1016/j.jad.2016.06.047 27367306

[8] Su Z Zou Z Hay SI Liu Y Li S Chen H Global, regional, and national time trends in mortality for congenital heart disease, 1990–2019: an age-period-cohort analysis for the global burden of disease 2019 study. EClinicalMedicine. (2022) 43:101249. 10.1016/j.eclinm.2021.101249 35059612 PMC8760503

[9] Bai Z Han J An J Wang H Du X Yang Z The global, regional, and national patterns of change in the burden of congenital birth defects, 1990–2021: an analysis of the global burden of disease study 2021 and forecast to 2040. EClinicalMedicine. (2024) 77:102873. 10.1016/j.eclinm.2024.102873 39416384 PMC11474384

[10] van der Linde D Konings EE Slager MA Witsenburg M Helbing WA Takkenberg JJ Birth prevalence of congenital heart disease worldwide: a systematic review and meta-analysis. J Am Coll Cardiol. (2011) 58:2241–7. 10.1016/j.jacc.2011.08.025 22078432

[11] United Nations Economic and Social Commission for Asia and the Pacific (ESCAP). Sustainable Development Goal 3: Ensure Healthy Lives and Promote Well-being for all Ages. Bangkok: United Nations Economic and Social Commission for Asia and the Pacific (2016). Available online at: https://repository.unescap.org/items/c5df0c8f-e7c1-4136-bc66-1a0adfcd32cf

[12] Chowdhury D Elliott PA Asaki SY Amdani S Nguyen QT Ronai C Addressing disparities in pediatric congenital heart disease: a call for equitable health care. J Am Heart Assoc. (2024) 13:e032415. 10.1161/JAHA.123.032415 38934870 PMC11255720

[13] Lopez KN Allen KY Baker-Smith CM Bravo-Jaimes K Burns J Cherestal B Health equity and policy considerations for pediatric and adult congenital heart disease care among minoritized populations in the United States. J Cardiovasc Dev Dis. (2024) 11(2):36. 10.3390/jcdd11020036 38392250 PMC10888593

[14] Reefhuis J Gilboa SM Anderka M Browne ML Feldkamp ML Hobbs CA The national birth defects prevention study: a review of the methods. Birth Defects Res A Clin Mol Teratol. (2015) 103:656–69. 10.1002/bdra.23384 26033852 PMC4558899

[15] Hjort L Møller SL Minja D Msemo O Nielsen BB Christensen DL FOETAL For NCD-FOetal exposure and epidemiological transitions: the role of anaemia in early life for non-communicable diseases in later life: a prospective preconception study in rural Tanzania. BMJ Open. (2019) 9(5):e024861. 10.1136/bmjopen-2018-024861 31122967 PMC6537995

[16] Lorente M Azpiroz MJ Guedes P Burgos R Lluch A Dos L. Nutrition, dietary recommendations, and supplements for patients with congenital heart disease. Int J Cardiol Congenit Heart Dis. (2023) 12:100449. 10.1016/j.ijcchd.2023.100449 39711821 PMC11657916

[17] Mao B Qiu J Zhao N Shao Y Dai W He X Maternal folic acid supplementation and dietary folate intake and congenital heart defects. PLoS One. (2017) 12:e0187996. 10.1371/journal.pone.0187996 29145433 PMC5690601

[18] Czeizel AE Dudás I Vereczkey A Bánhidy F. Folate deficiency and folic acid supplementation: the prevention of neural-tube defects and congenital heart defects. Nutrients. (2013) 5(11):4760–75. 10.3390/nu5114760 24284617 PMC3847759

[19] Scott-Goodwin AC Puerto M Moreno I. Toxic effects of prenatal exposure to alcohol, tobacco and other drugs. Reprod Toxicol. (2016) 61:120–30. 10.1016/j.reprotox.2016.03.043 27037188

[20] Feng Y Yu D Yang L Da M Wang Z Lin Y Maternal lifestyle factors in pregnancy and congenital heart defects in offspring: review of the current evidence. Ital J Pediatr. (2014) 40:85. 10.1186/s13052-014-0085-3 25385357 PMC4243331

[21] GBD 2021 Diseases and Injuries Collaborators. Global incidence, prevalence, years lived with disability (YLDs), disability-adjusted life-years (DALYs), and healthy life expectancy (HALE) for 371 diseases and injuries in 204 countries and territories and 811 subnational locations, 1990–2021: a systematic analysis for the global burden of disease study 2021. Lancet. (2024) 403(10440):2133–61. 10.1016/S0140-6736(24)00757-8 38642570 PMC11122111

[22] Apers S Kovacs AH Luyckx K Thomet C Budts W Enomoto J Quality of life of adults with congenital heart disease in 15 countries: evaluating country-specific characteristics. J Am Coll Cardiol. (2016) 67(19):2237–45. 10.1016/j.jacc.2016.03.477 27173035

[23] Best KE Rankin J. Long-term survival of individuals born with congenital heart disease: a systematic review and meta-analysis. J Am Heart Assoc. (2016) 5(6):e002120. 10.1161/JAHA.116.002120 27312802 PMC4937249

[24] Roth GA Mensah GA Johnson CO Addolorato G Ammirati E Baddour LM Global burden of cardiovascular diseases and risk factors, 1990–2019: update from the GBD 2019 study. J Am Coll Cardiol. (2021) 77(15):1958–9. 10.1016/j.jacc.2021.02.039 33309175 PMC7755038

[25] Kenny DP Hijazi ZM. Current status and future potential of transcatheter interventions in congenital heart disease. Circ Res. (2017) 120(6):1015–26. 10.1161/CIRCRESAHA.116.309185 28302745

[26] Zühlke L Lawrenson J Comitis G De Decker R Brooks A Fourie B Congenital heart disease in low- and lower-middle-income countries: current status and new opportunities. Curr Cardiol Rep. (2019) 21(12):163. 10.1007/s11886-019-1248-z 31784844

[27] Louis JD S Kurosawa H Jonas RA Sandoval N Cervantes J Tchervenkov CI The world database for pediatric and congenital heart surgery: the Dawn of a new era of global communication and quality improvement in congenital heart disease. World J Pediatr Congenit Heart Surg. (2017) 8(5):597–9. 10.1177/2150135117725458 28901228

[28] Su Z Zhang Y Cai X Li Q Gu H Luan Y Improving long-term care and outcomes of congenital heart disease: fulfilling the promise of a healthy life. Lancet Child Adolesc Health. (2023) 7(7):502–18. 10.1016/S2352-4642(23)00053-6 37301214

[29] Salerni S Di Francescomarino S Cadeddu C Acquistapace F Maffei S Gallina S. The different role of sex hormones on female cardiovascular physiology and function: not only oestrogens. Eur J Clin Invest. (2015) 45(6):634–45. 10.1111/eci.12447 25845675

[30] Iorga A Cunningham CM Moazeni S Ruffenach G Umar S Eghbali M. The protective role of estrogen and estrogen receptors in cardiovascular disease and the controversial use of estrogen therapy. Biol Sex Differ. (2017) 8(1):33. 10.1186/s13293-017-0152-8 29065927 PMC5655818

[31] de Marvao A Alexander D Bucciarelli-Ducci C Price S. Heart disease in women: a narrative review. Anaesthesia. (2021) 76(Suppl 4):118–30. 10.1111/anae.15376 33682102

[32] Davey B Sinha R Lee JH Gauthier M Flores G. Social determinants of health and outcomes for children and adults with congenital heart disease: a systematic review. Pediatr Res. (2021) 89(2):275–94. 10.1038/s41390-020-01196-6 33069160

[33] Chowdhury D Johnson JN Baker-Smith CM Jaquiss RDB Mahendran AK Curren V Health care policy and congenital heart disease: 2020 focus on our 2030 future. J Am Heart Assoc. (2021) 10(20):e020605. 10.1161/JAHA.120.020605 34622676 PMC8751886

[34] Baumgartner H De Backer J Babu-Narayan SV Budts W Chessa M Diller GP 2020 ESC guidelines for the management of adult congenital heart disease. Eur Heart J. (2021) 42(6):563–645. 10.1093/eurheartj/ehaa554 32860028

[35] Yoon SA Hong WH Cho HJ. Congenital heart disease diagnosed with echocardiogram in newborns with asymptomatic cardiac murmurs: a systematic review. BMC Pediatr. (2020) 20(1):322. 10.1186/s12887-020-02212-8 32605548 PMC7325562

[36] United Nations, Department of Economic and Social Affairs, Population Division. World Population Prospects 2022: Summary of Results. UN DESA/POP/2022/TR/NO. 3. New York: United Nations (2022). Available online at: https://www.un.org/development/desa/pd/sites/www.un.org.development.desa.pd/files/wpp2022_summary_of_results.pdf

[37] GBD 2019 Stroke Collaborators. Global, regional, and national burden of stroke and its risk factors, 1990-2019: a systematic analysis for the Global Burden of Disease Study 2019. Lancet Neurol. (2021) 20(10):795–820. 10.1016/S1474-4422(21)00252-0 34487721 PMC8443449

[38] Wafa HA Marshall I Wolfe CDA Xie W Johnson CO Veltkamp R Burden of intracerebral haemorrhage in Europe: forecasting incidence and mortality between 2019 and 2050. Lancet Reg Health Eur. (2024) 38:100842. 10.1016/j.lanepe.2024.100842 38362494 PMC10867656

[39] Foreman KJ Lozano R Lopez AD Murray CJ. Modeling causes of death: an integrated approach using CODEm. Popul Health Metr. (2012) 10:1. 10.1186/1478-7954-10-1 22226226 PMC3315398

[40] Møller B Fekjaer H Hakulinen T Sigvaldason H Storm HH Talbäck M Prediction of cancer incidence in the nordic countries: empirical comparison of different approaches. Stat Med. (2003) 22(17):2751–66. 10.1002/sim.1481 12939784

