# Supplementary material for: Global, regional, and national burdens of congenital heart anomalies from 1990 to 2021, and projections to 2050
Source: Front Pediatr. 2025 Aug 18;13:1601620. doi: 10.3389/fped.2025.1601620 (PMC12399661; doi:10.3389/fped.2025.1601620)
Supplement: Supplementary file 2 [file Datasheet1.pdf]

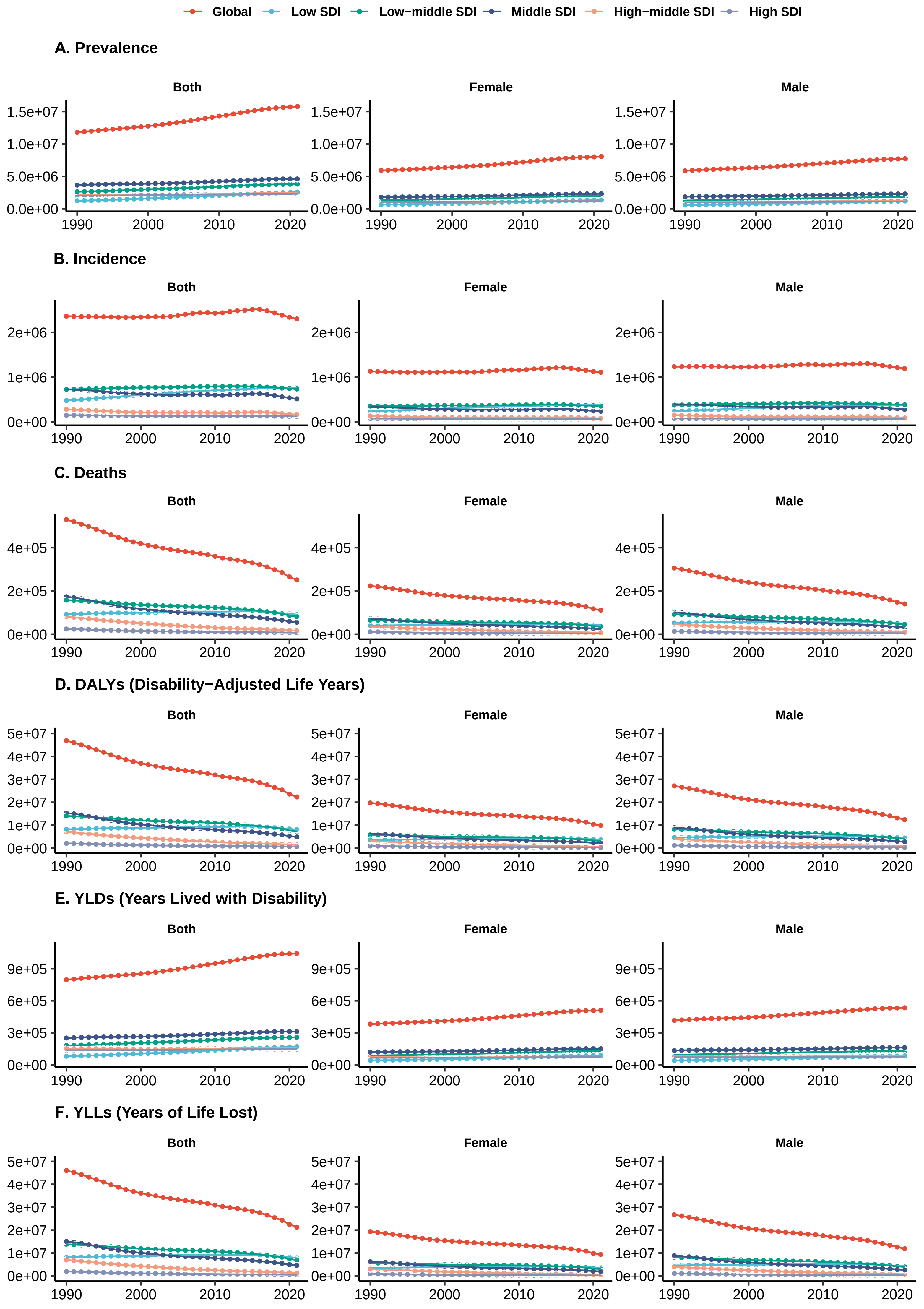
Global
Low SDI
Low−middle SDI
Middle SDI
High−middle SDI
High SDI
A. Prevalence
Both
Female
Male
0.0e+00
5.0e+06
1.0e+07
1.5e+07
1990
2000
2010
2020
B. Incidence
Both
Female
Male
0e+00
1e+06
2e+06
1990
2000
2010
2020
C. Deaths
Both
Female
Male
0e+00
2e+05
4e+05
1990
2000
2010
2020
D. DALYs (Disability−Adjusted Life Years)
Both
Female
Male
0e+00
1e+07
2e+07
3e+07
4e+07
5e+07
1990
2000
2010
2020
E. YLDs (Years Lived with Disability)
Both
Female
Male
0e+00
3e+05
6e+05
9e+05
1990
2000
2010
2020
F. YLLs (Years of Life Lost)
Both
Female
Male
0e+00
1e+07
2e+07
3e+07
4e+07
5e+07
1990
2000
2010
2020
