# Supplementary figures and images for: Global, regional, and national burdens of congenital heart anomalies from 1990 to 2021, and projections to 2050
Source: Front Pediatr. 2025 Aug 18;13:1601620. doi: 10.3389/fped.2025.1601620 (PMC12399661; doi:10.3389/fped.2025.1601620)

Female Male

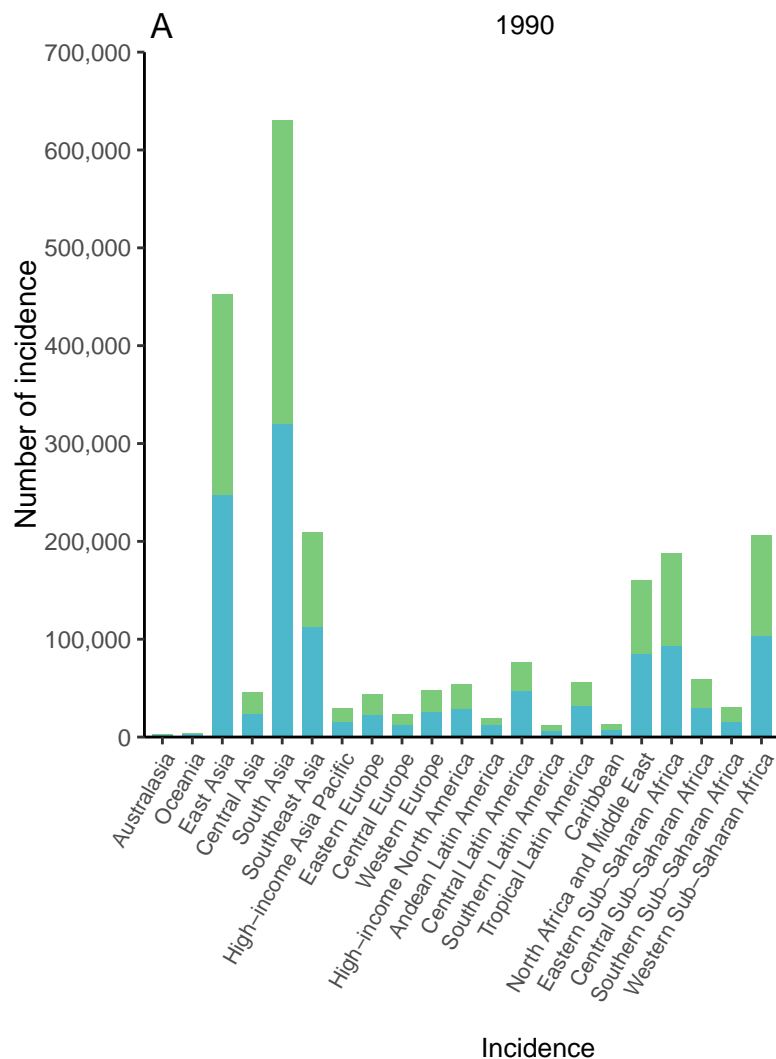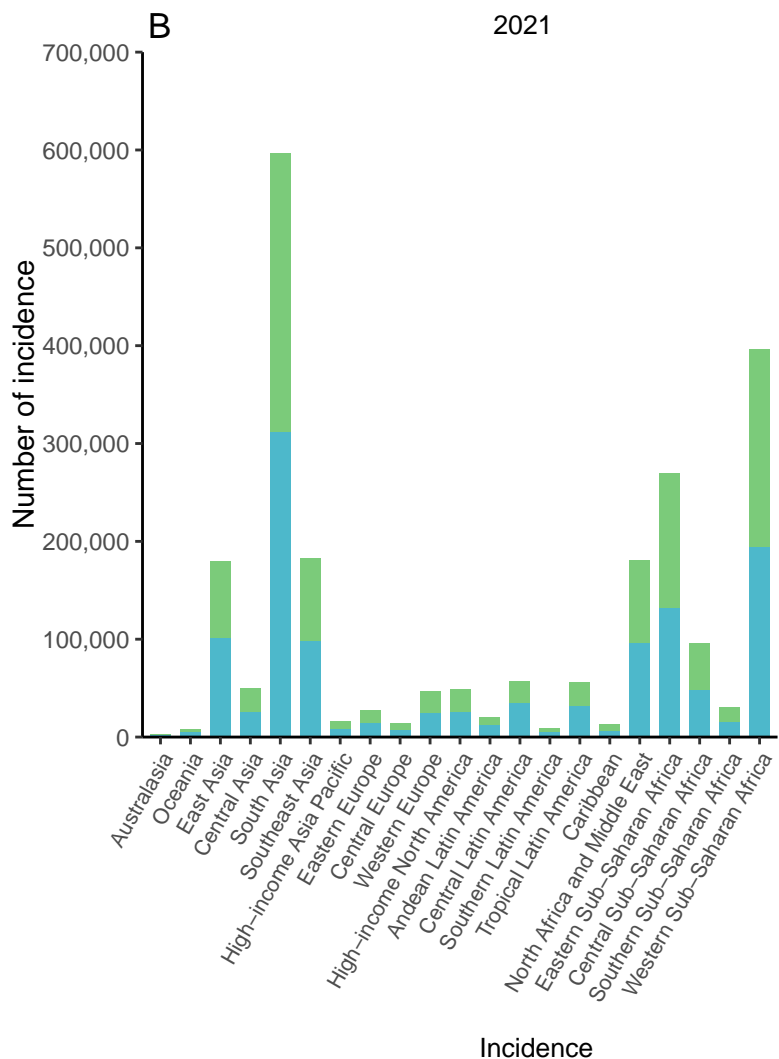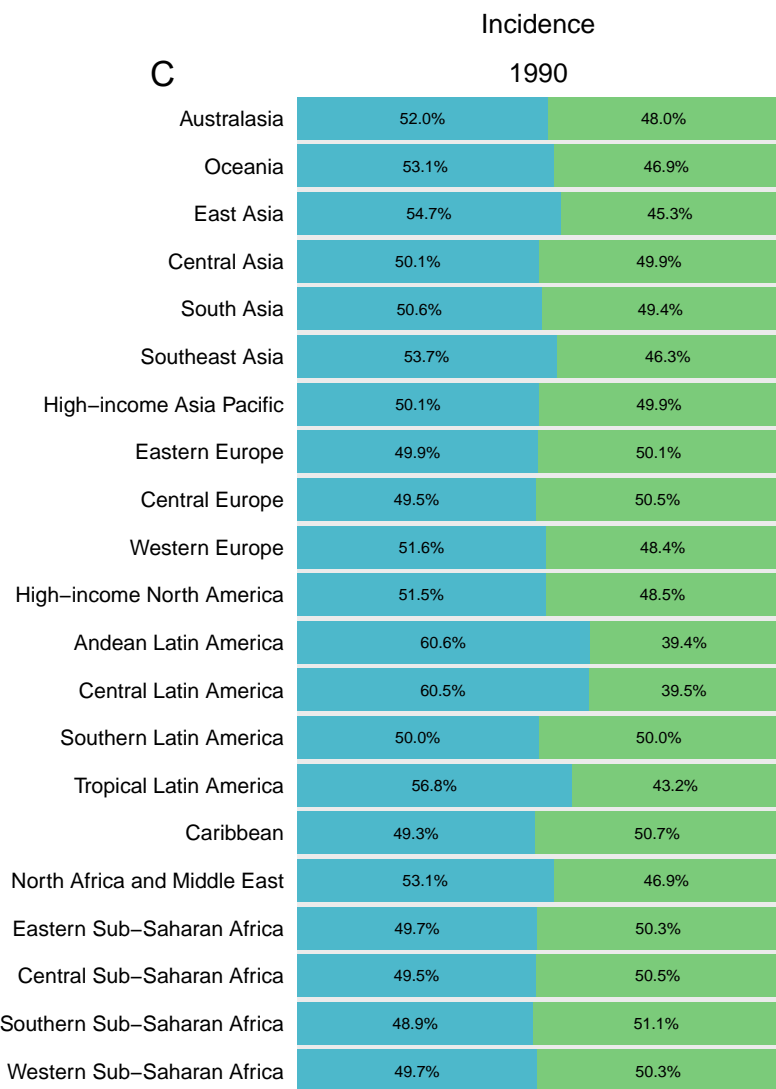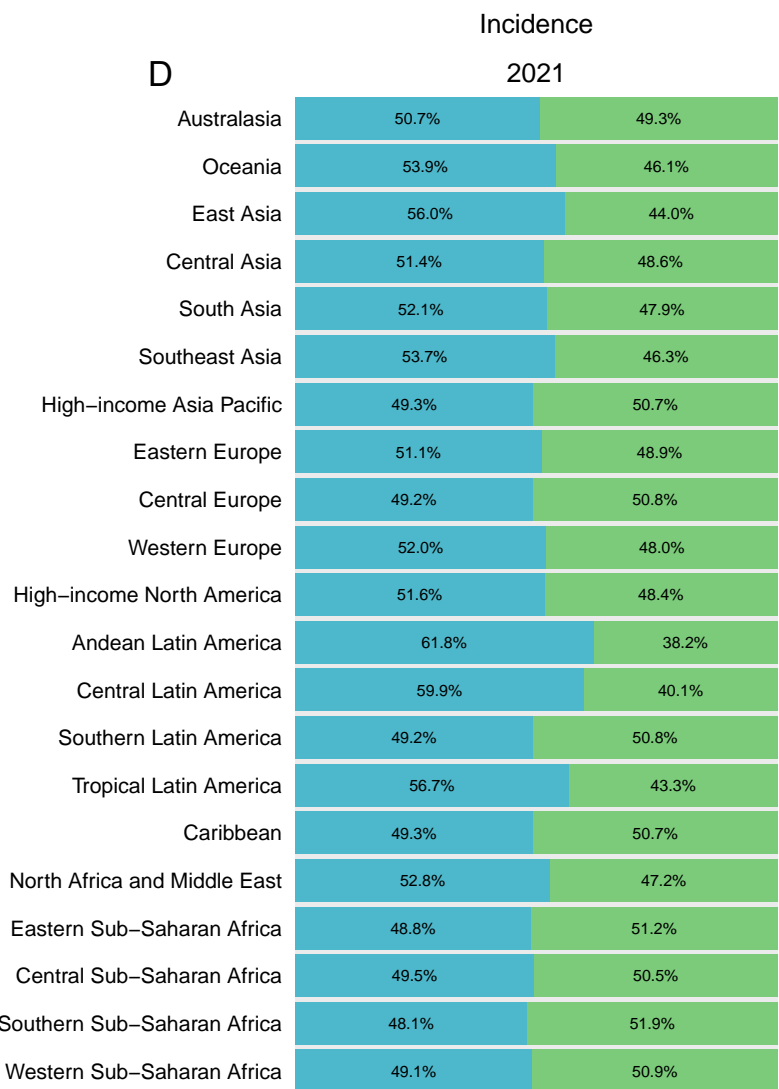

Supplement: Supplementary file 3 [file Datasheet2.pdf]

Female Male

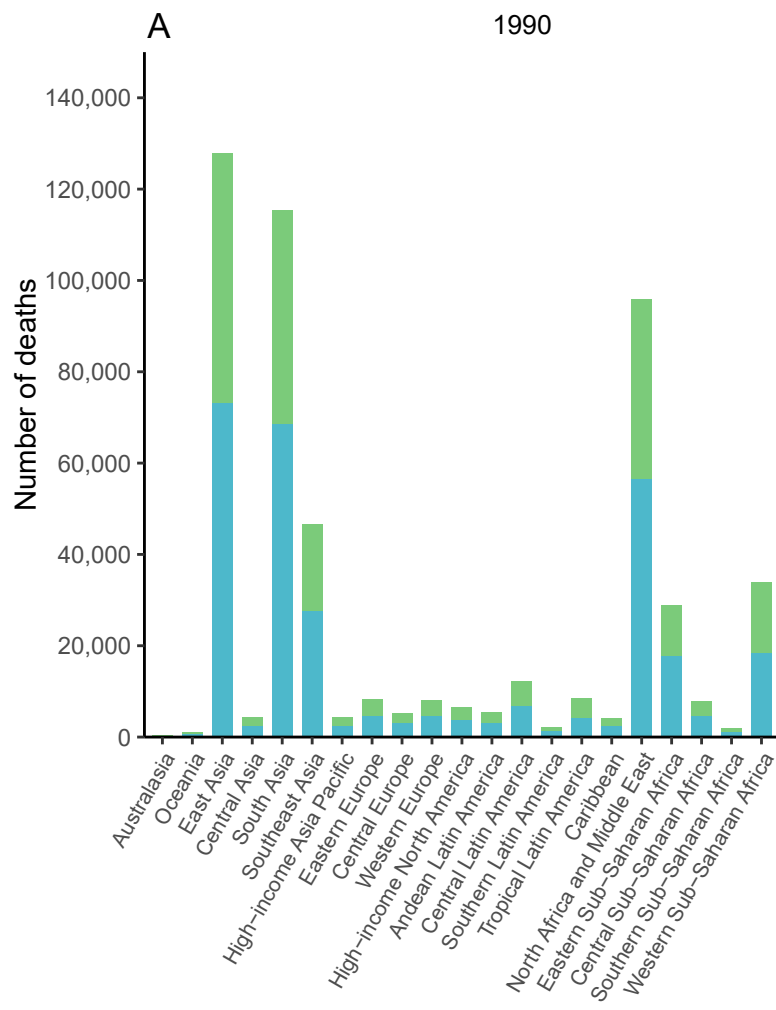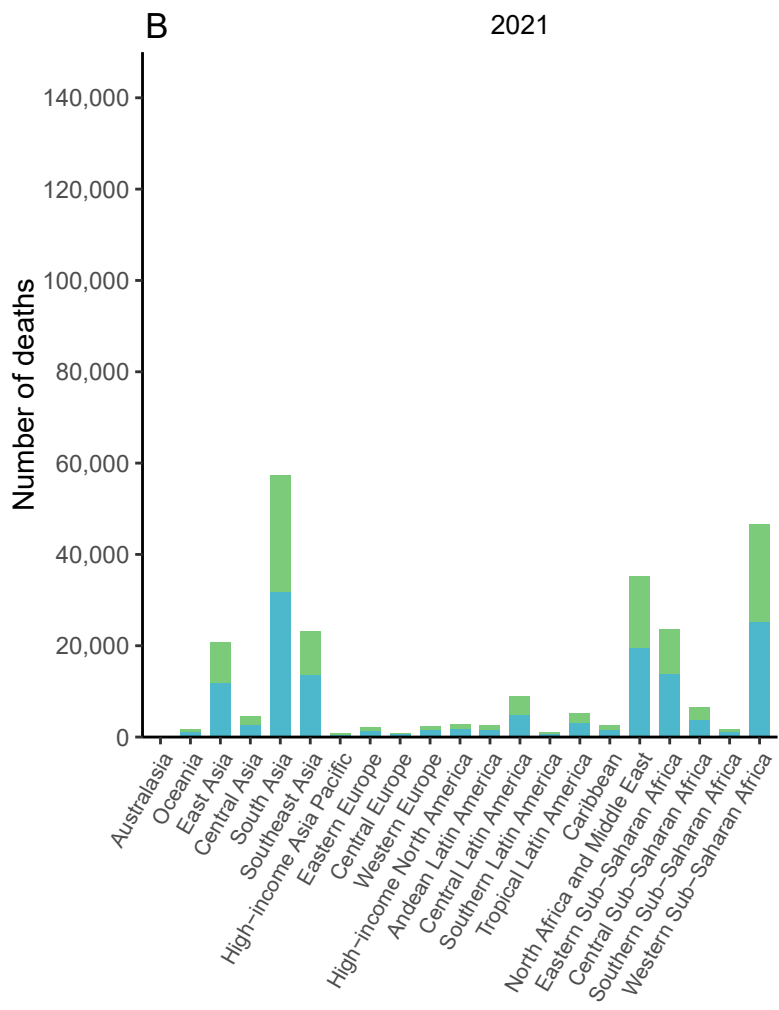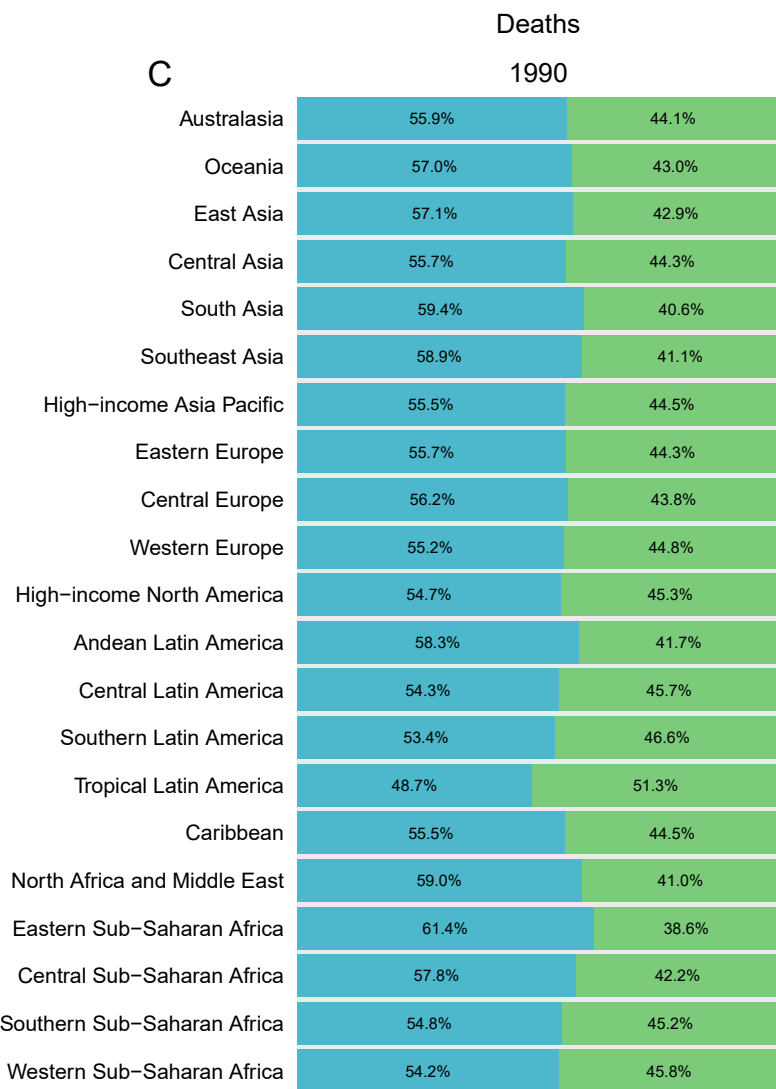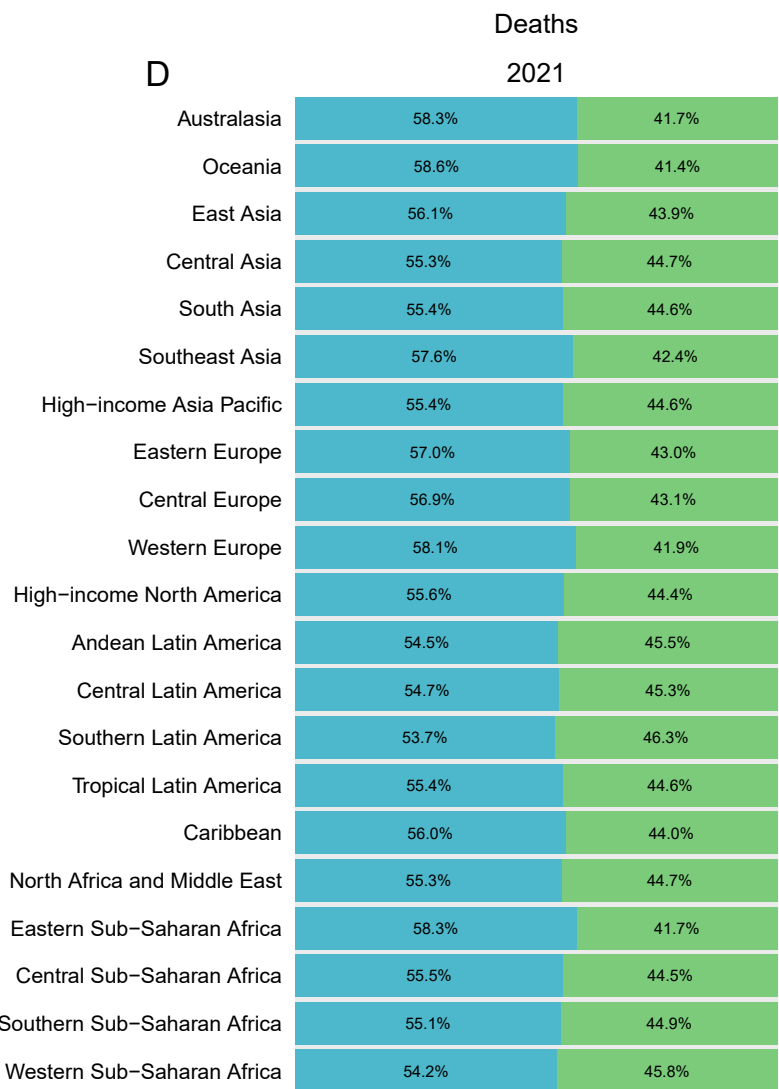

Supplement: Supplementary file 4 [file Datasheet3.pdf]

Female Male

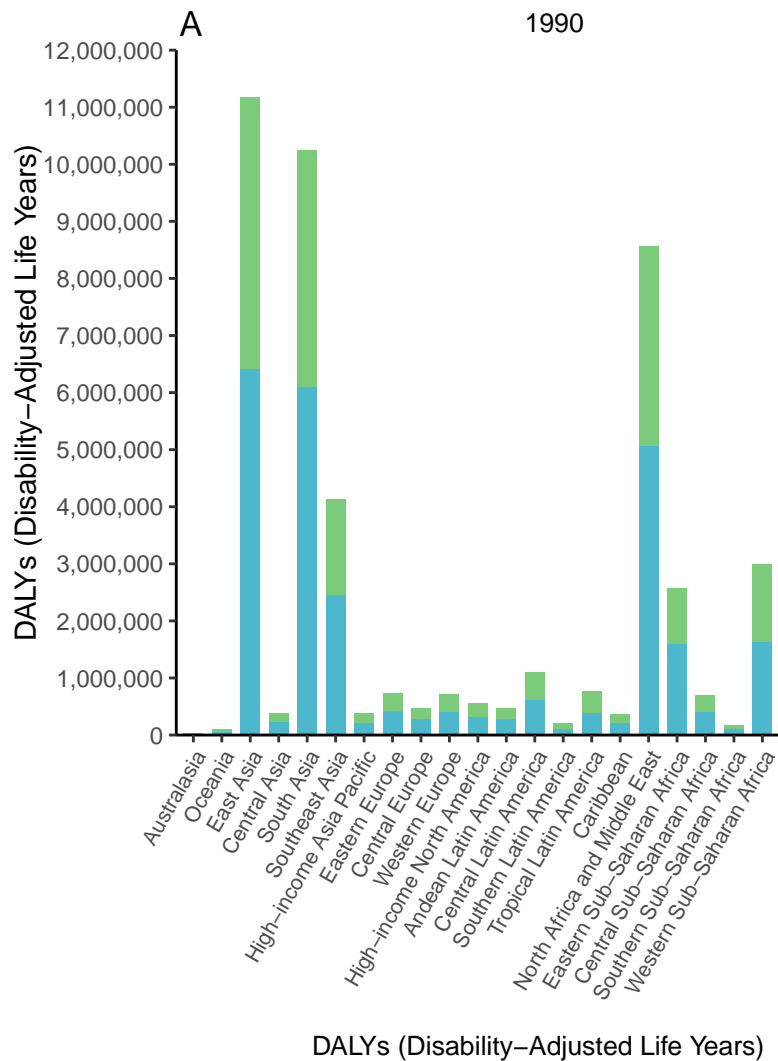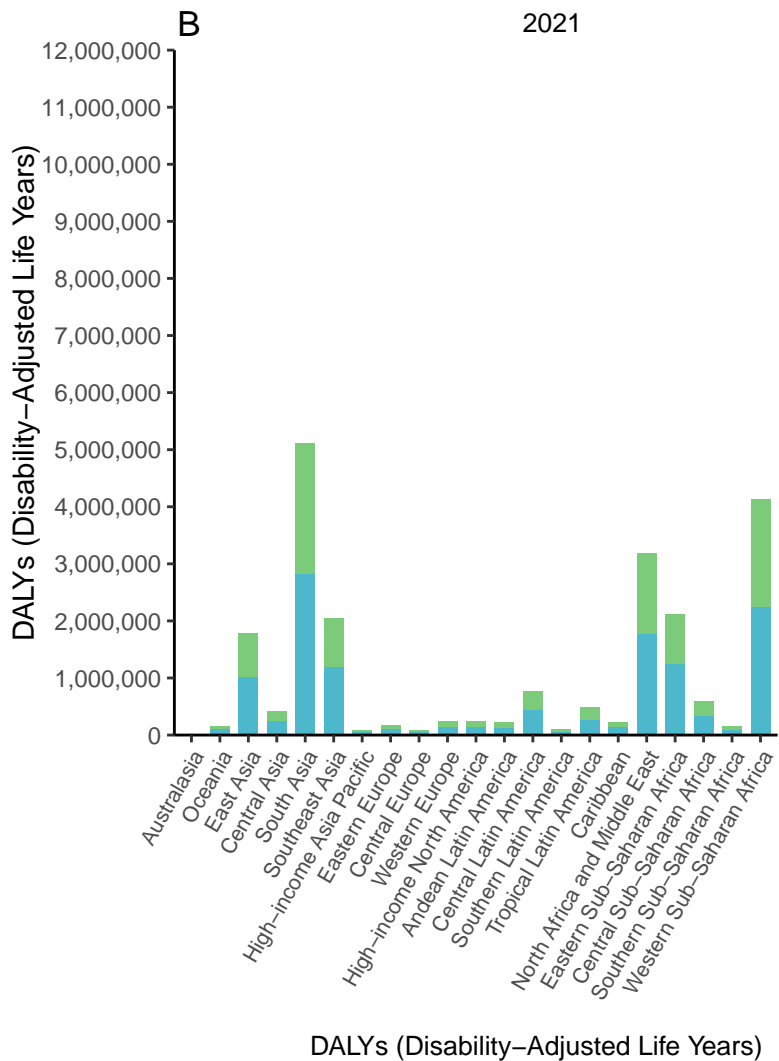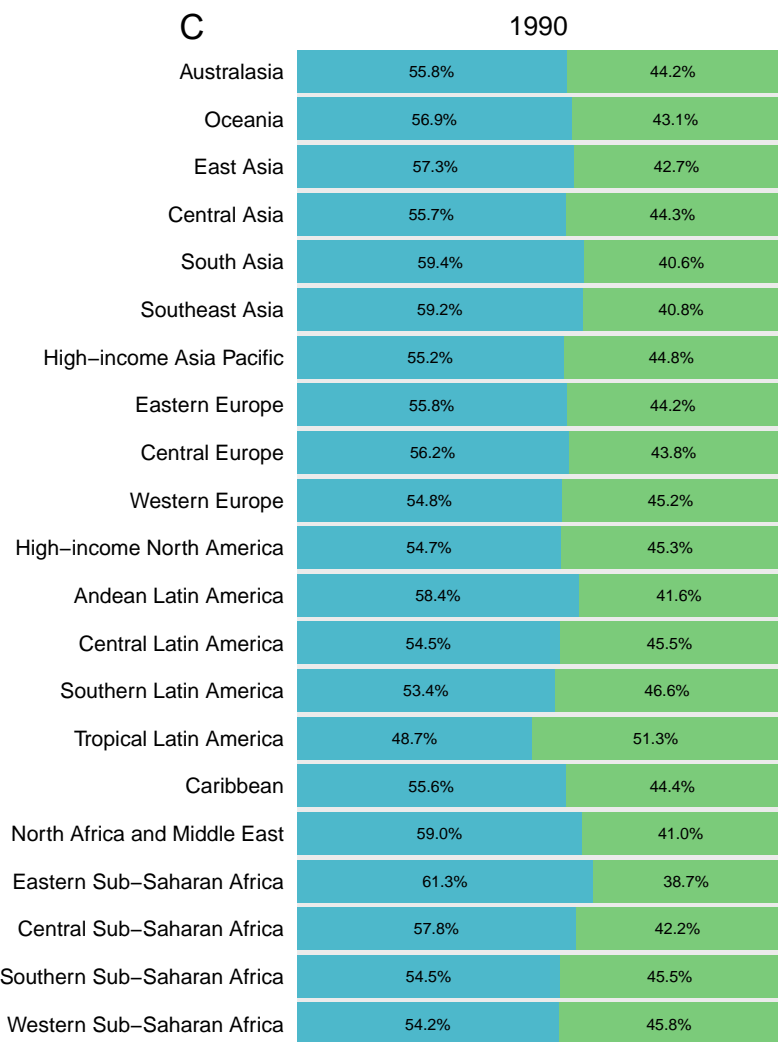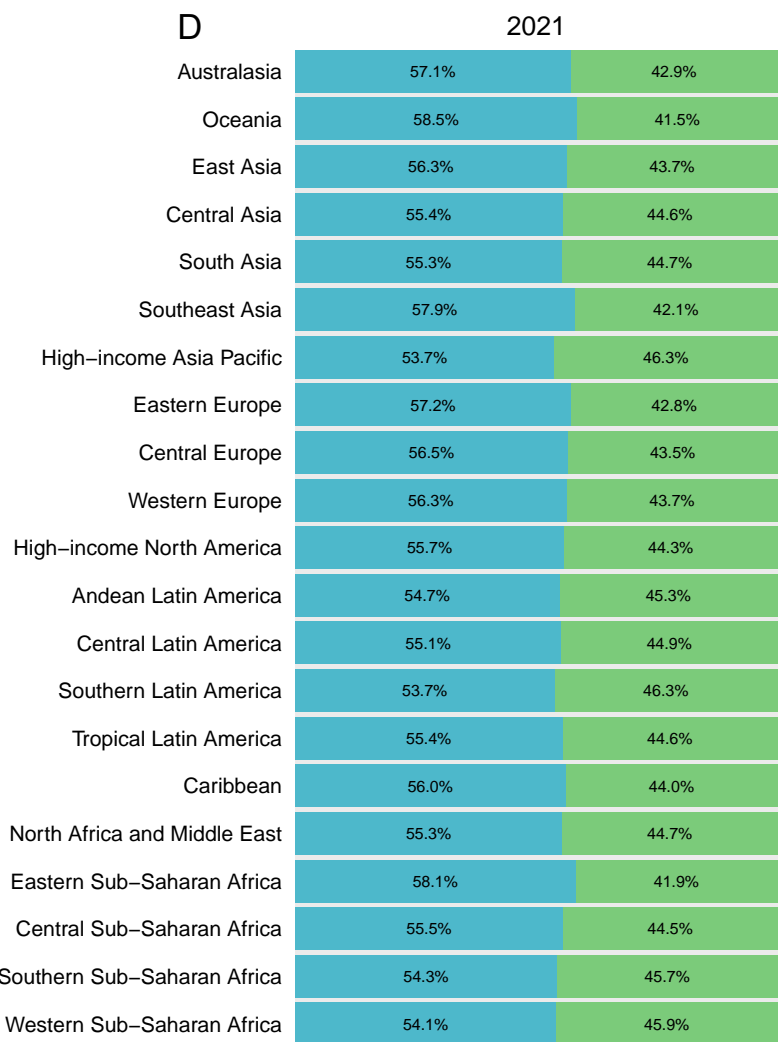

Supplement: Supplementary file 5 [file Datasheet4.pdf]

Female Male

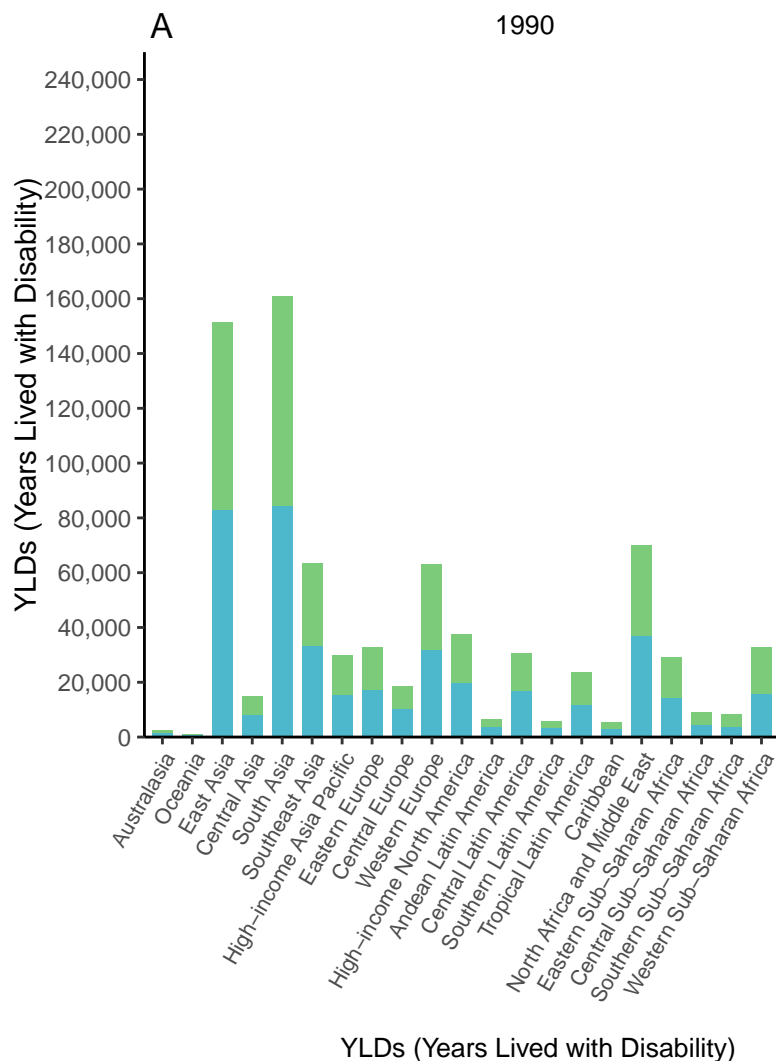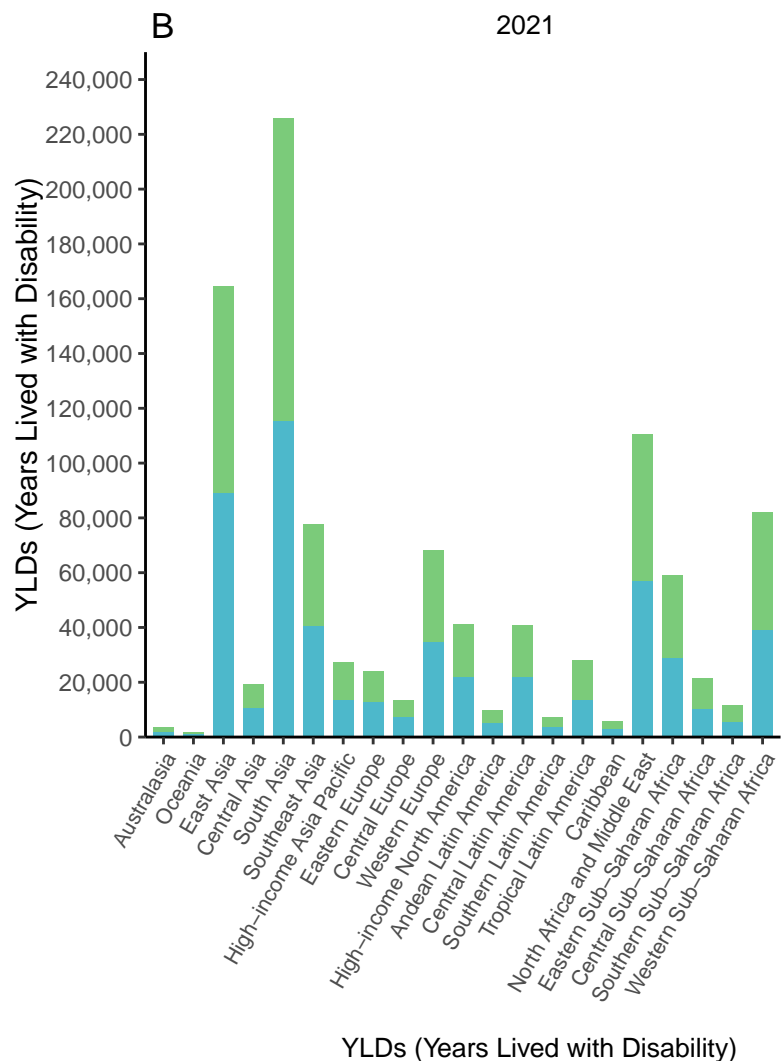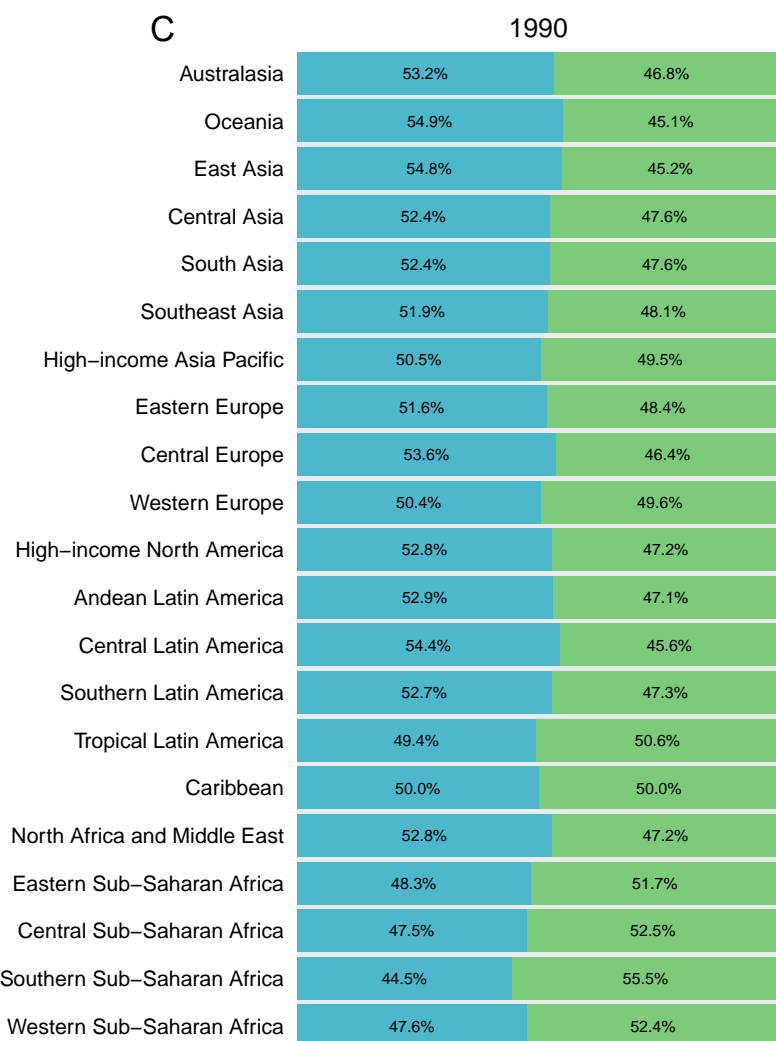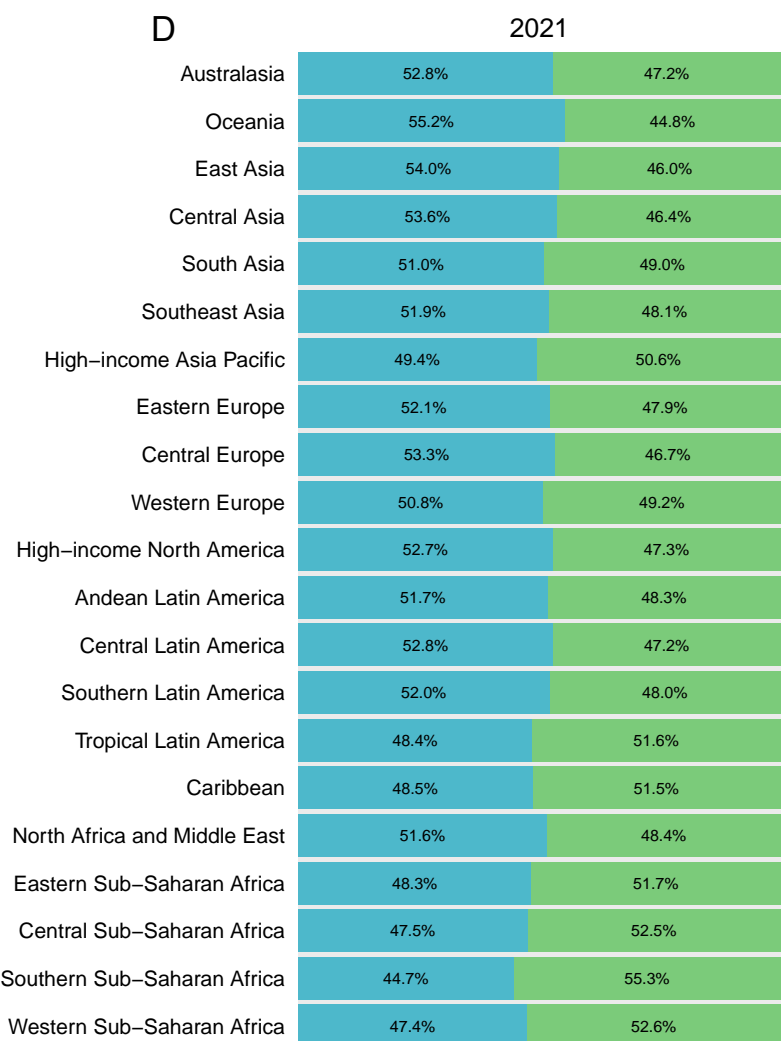

Supplement: Supplementary file 6 [file Datasheet5.pdf]

Female Male

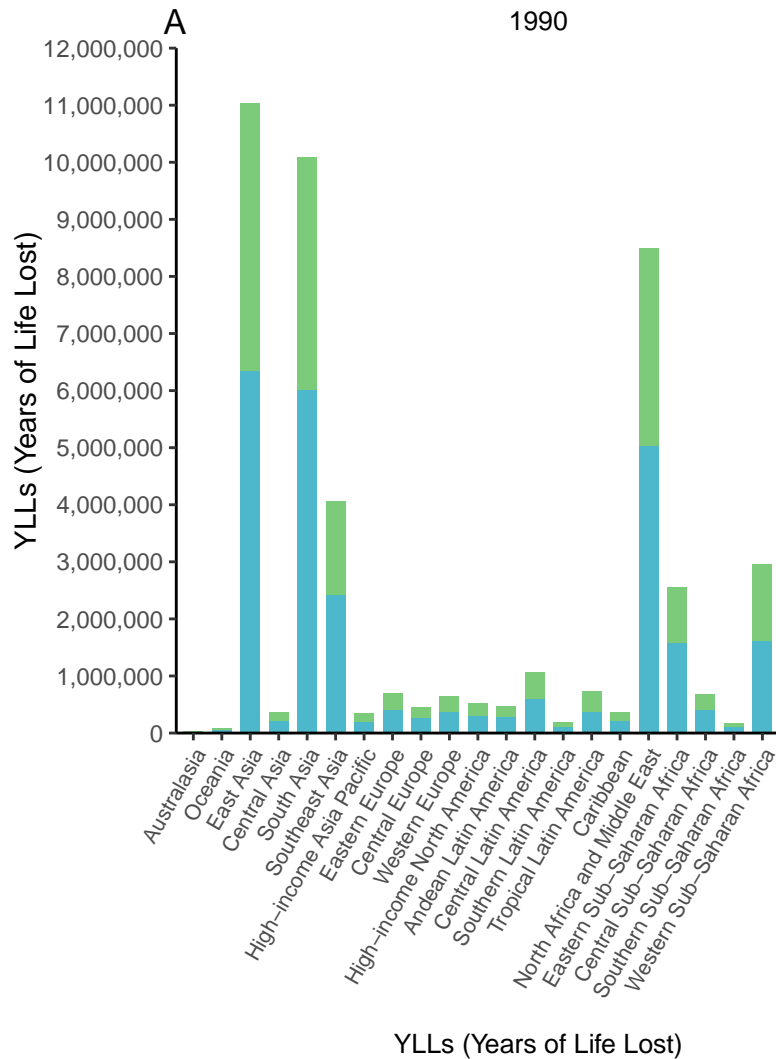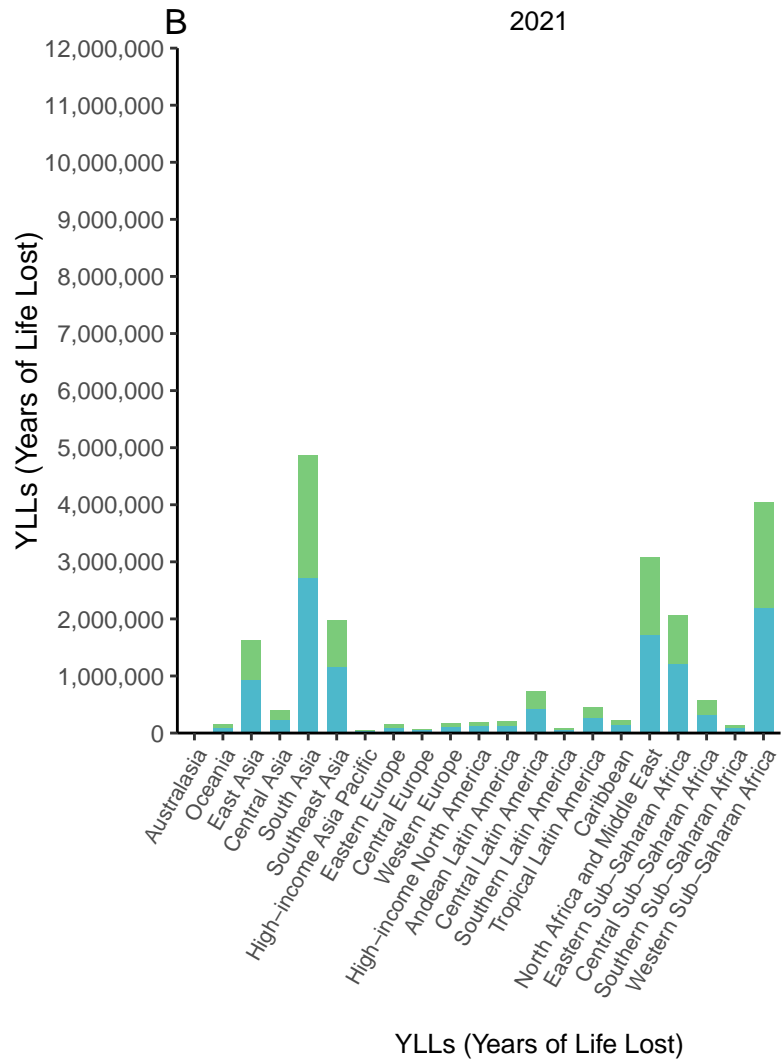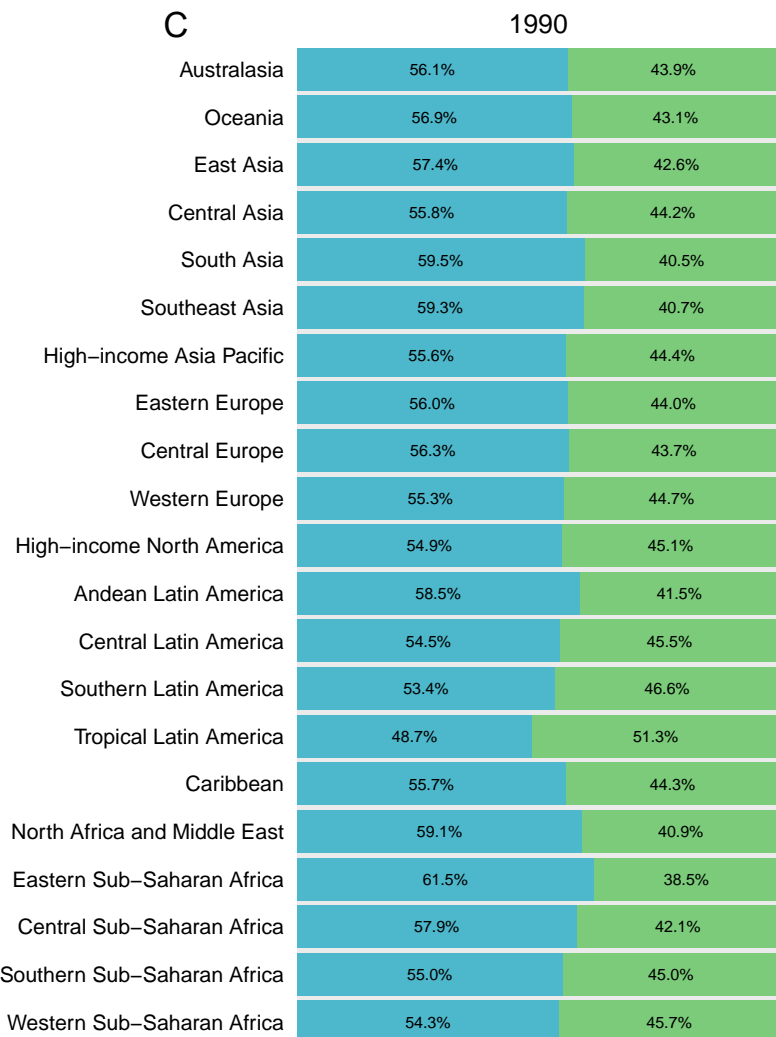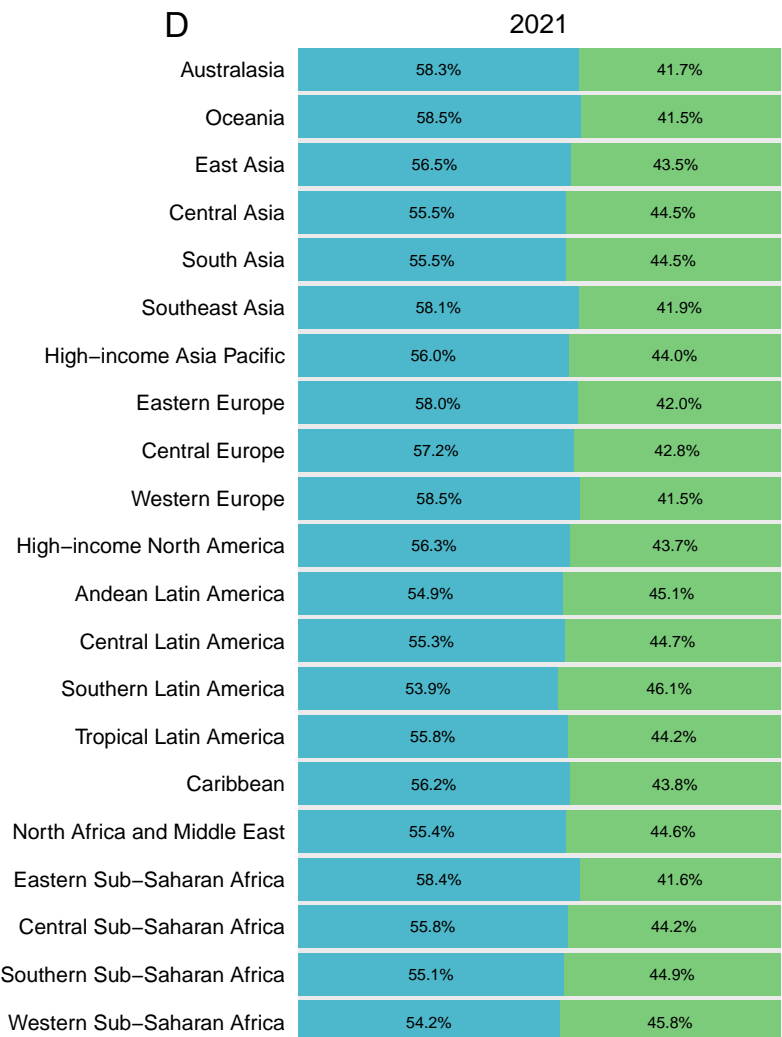

Supplement: Supplementary file 7 [file Datasheet6.pdf]

A

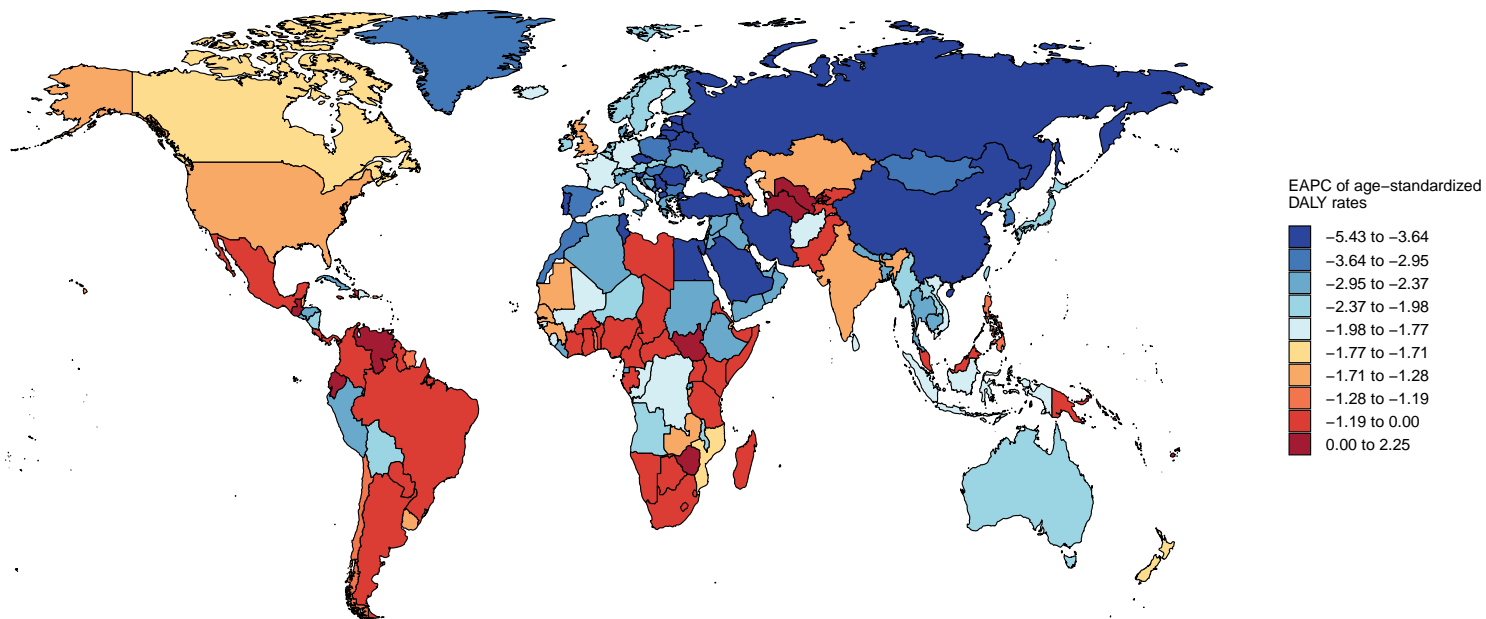

B

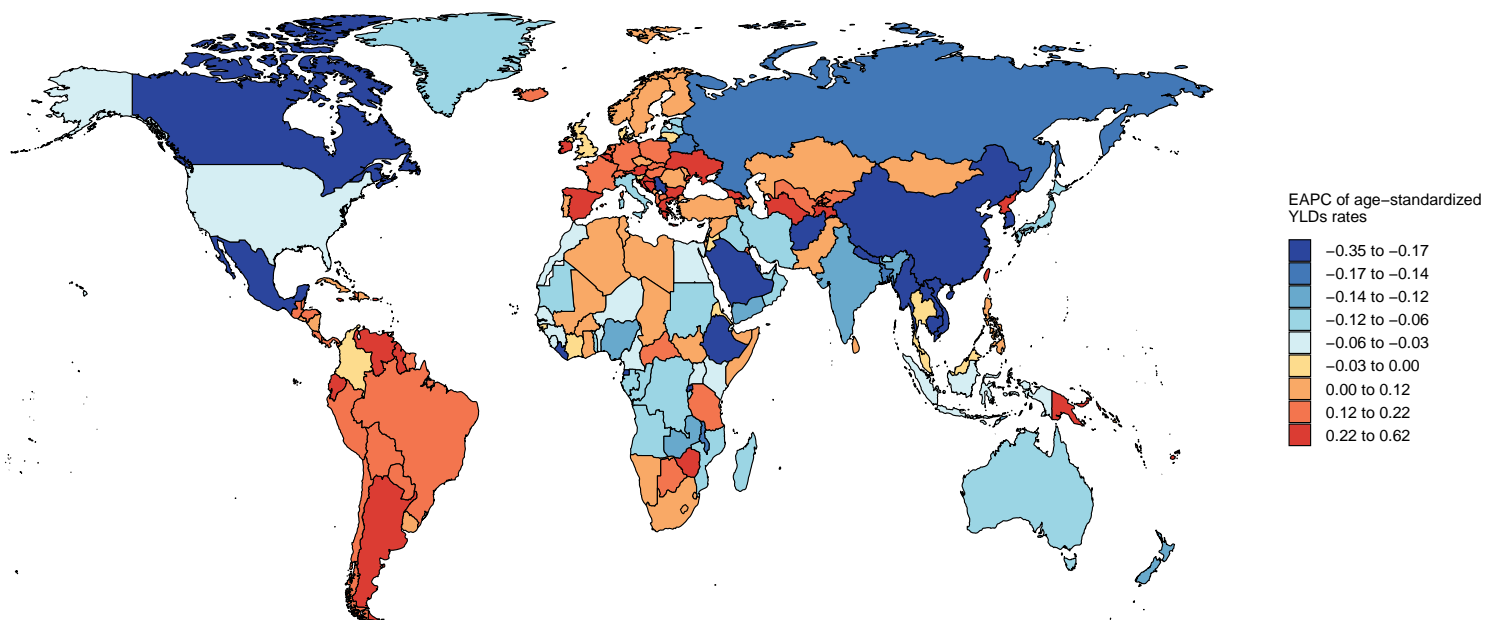

C

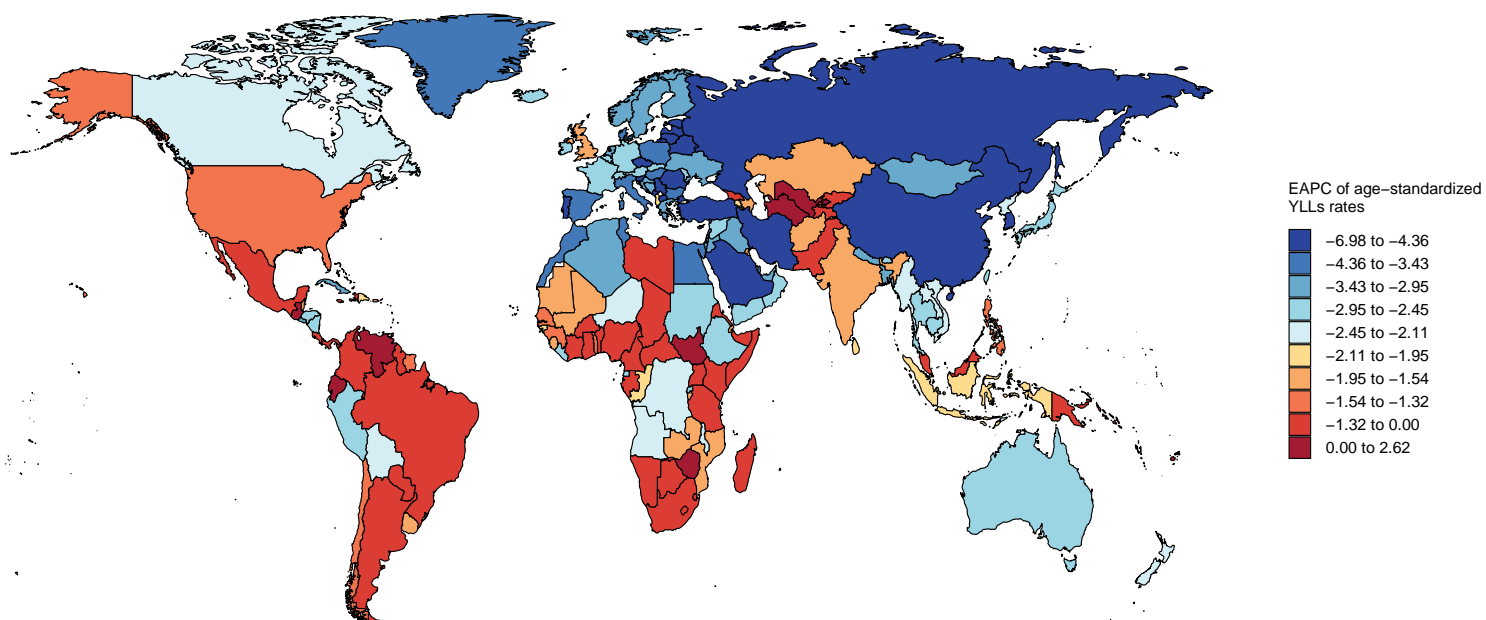

Supplement: Supplementary file 8 [file Datasheet7.pdf]

$$r = -0.5936, p < 0.001$$
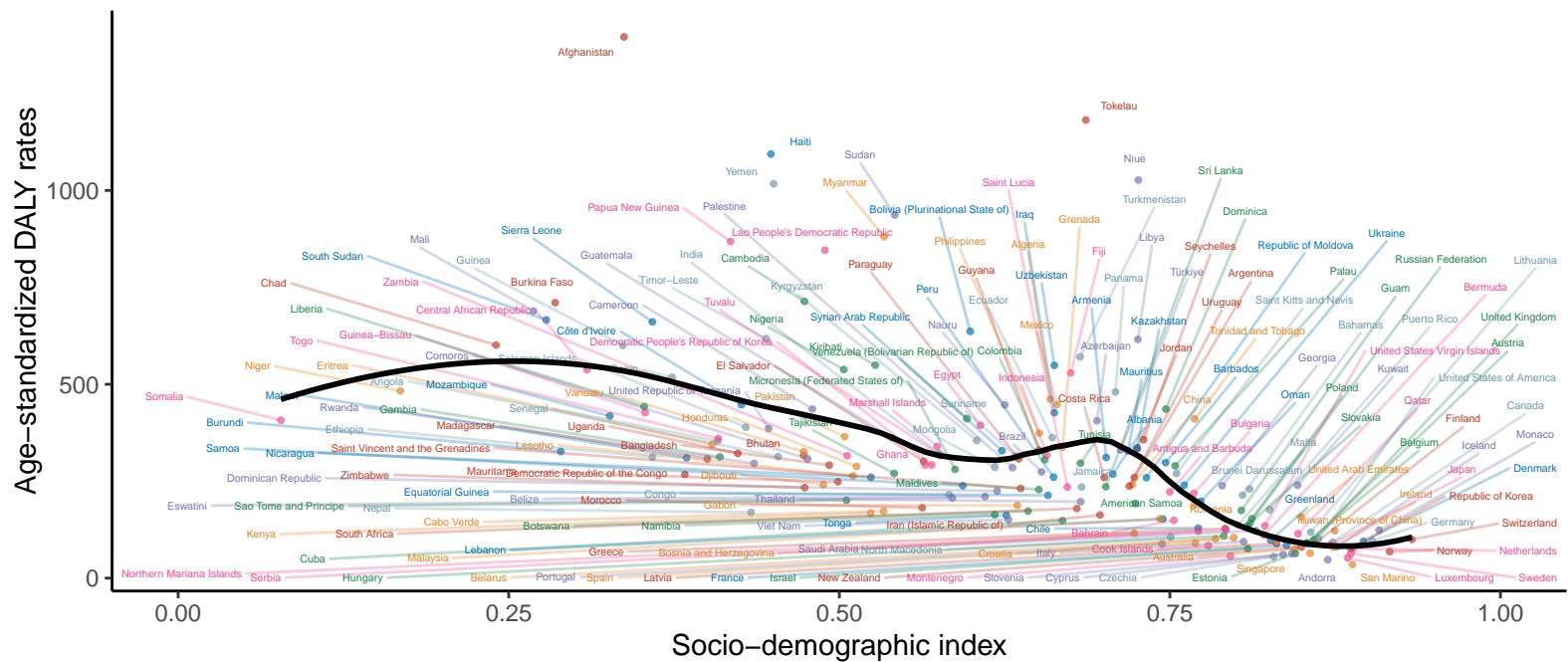

**B**

$$r = 0.6456 \quad p < 0.001$$
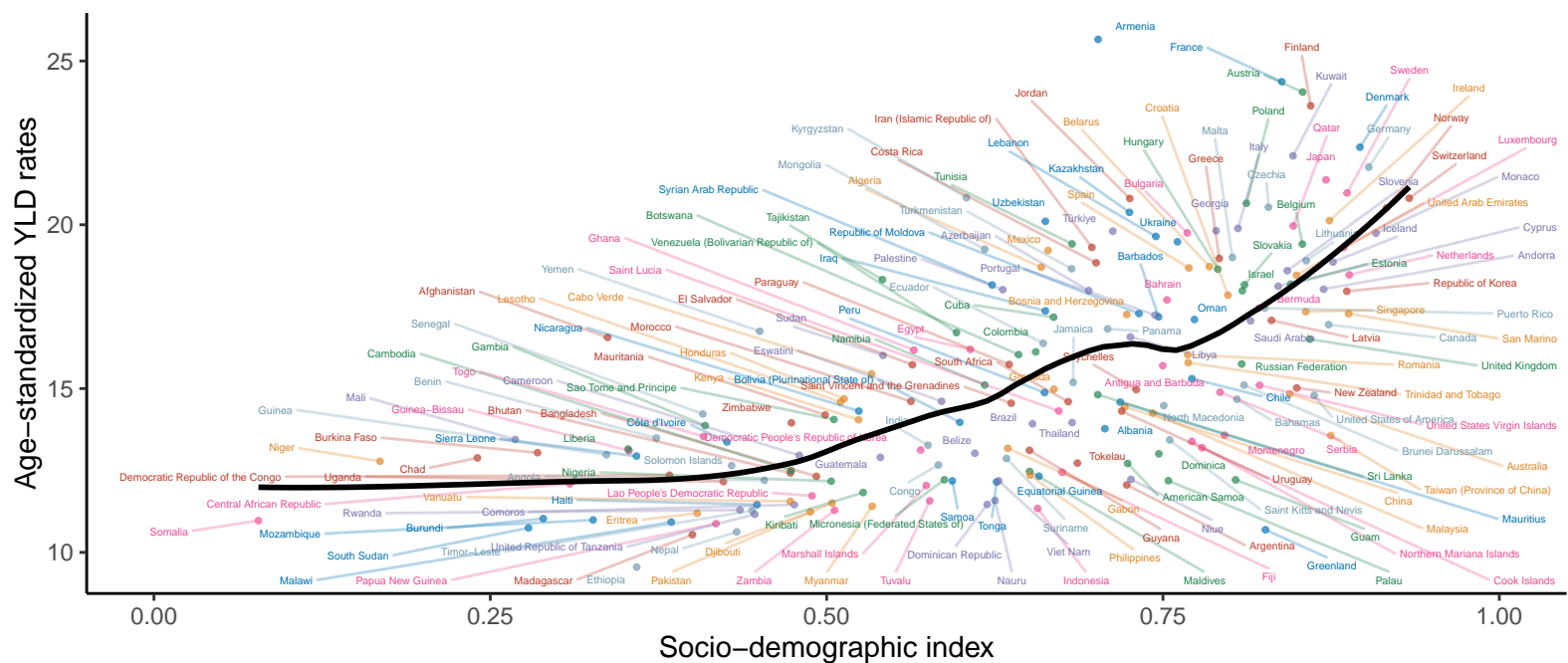

C

$$r = -0.5999, p < 0.001$$
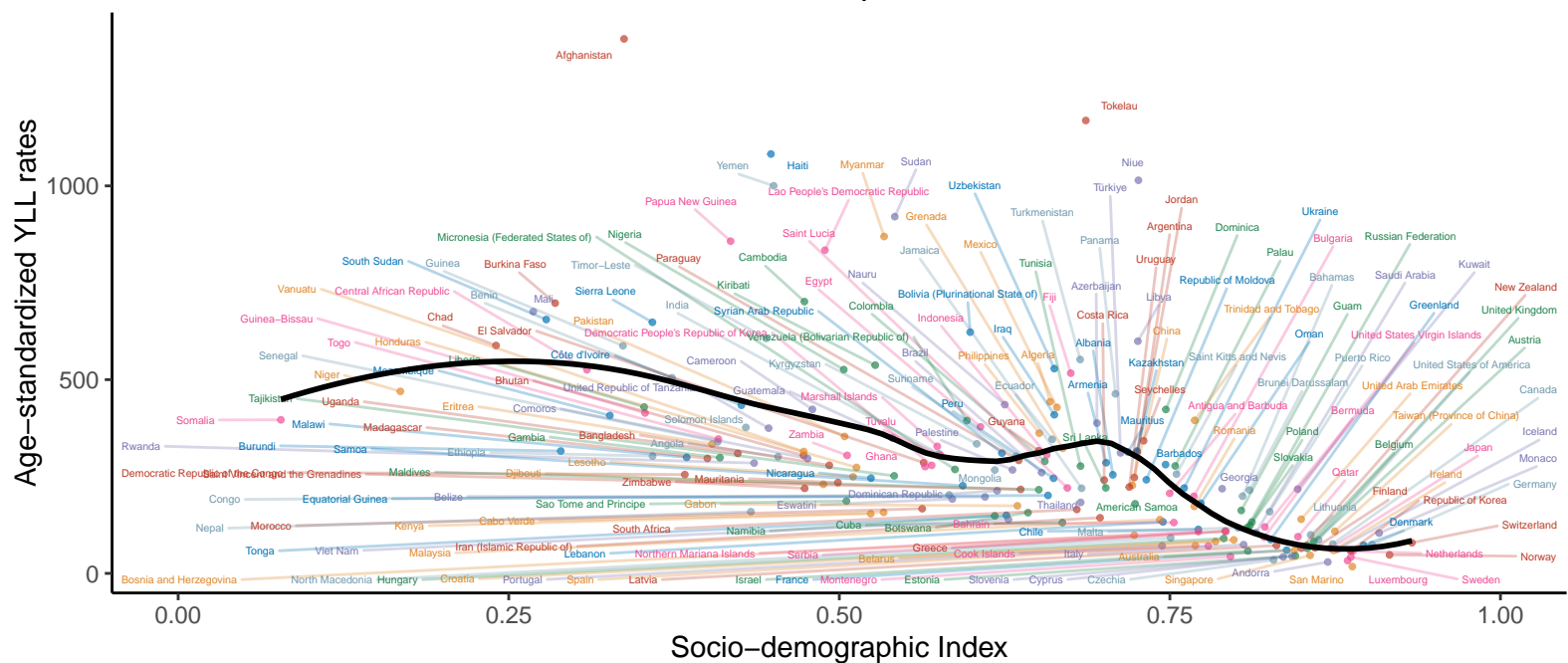

Supplement: Supplementary file 10 [file Datasheet9.pdf]
