# Supplementary material for: Global, regional, and national burdens of congenital heart anomalies from 1990 to 2021, and projections to 2050
Source: Front Pediatr. 2025 Aug 18;13:1601620. doi: 10.3389/fped.2025.1601620 (PMC12399661; doi:10.3389/fped.2025.1601620)
Supplement: Supplementary file 9 [file Datasheet8.pdf]

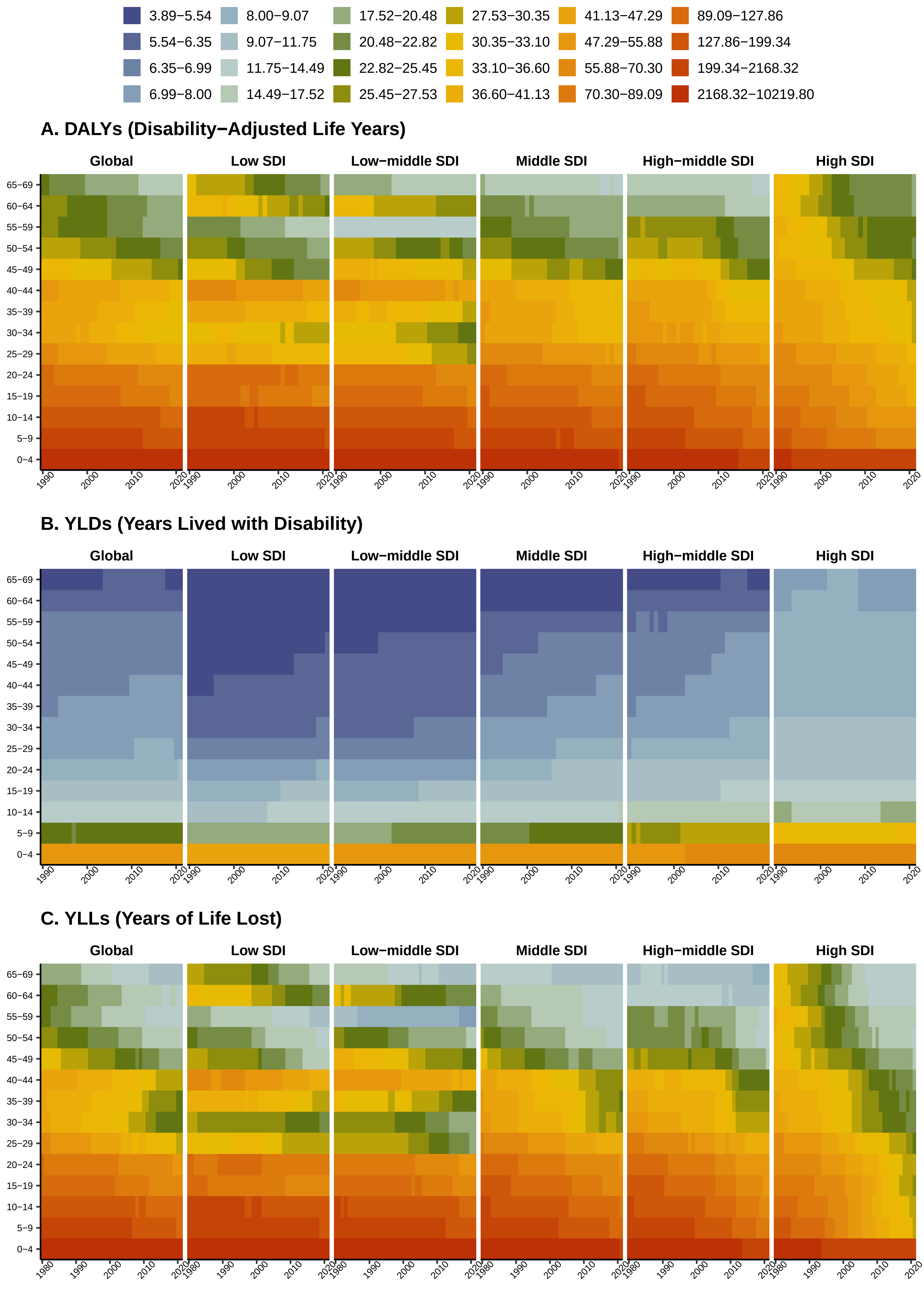
3.89−5.54
5.54−6.35
6.35−6.99
6.99−8.00
8.00−9.07
9.07−11.75
11.75−14.49
14.49−17.52
17.52−20.48
20.48−22.82
22.82−25.45
25.45−27.53
27.53−30.35
30.35−33.10
33.10−36.60
36.60−41.13
41.13−47.29
47.29−55.88
55.88−70.30
70.30−89.09
89.09−127.86
127.86−199.34
199.34−2168.32
2168.32−10219.80
A. DALYs (Disability−Adjusted Life Years)
Global
Low SDI
Low−middle SDI
Middle SDI
High−middle SDI
High SDI
65−69
60−64
55−59
50−54
45−49
40−44
35−39
30−34
25−29
20−24
15−19
10−14
5−9
0−4
1990
2000
2010
2020
B. YLDs (Years Lived with Disability)
C. YLLs (Years of Life Lost)
1980
