# Supplementary material for: Global, regional, and national burdens of congenital heart anomalies from 1990 to 2021, and projections to 2050
Source: Front Pediatr. 2025 Aug 18;13:1601620. doi: 10.3389/fped.2025.1601620 (PMC12399661; doi:10.3389/fped.2025.1601620)
Supplement: Supplementary file 11 [file Datasheet10.pdf]

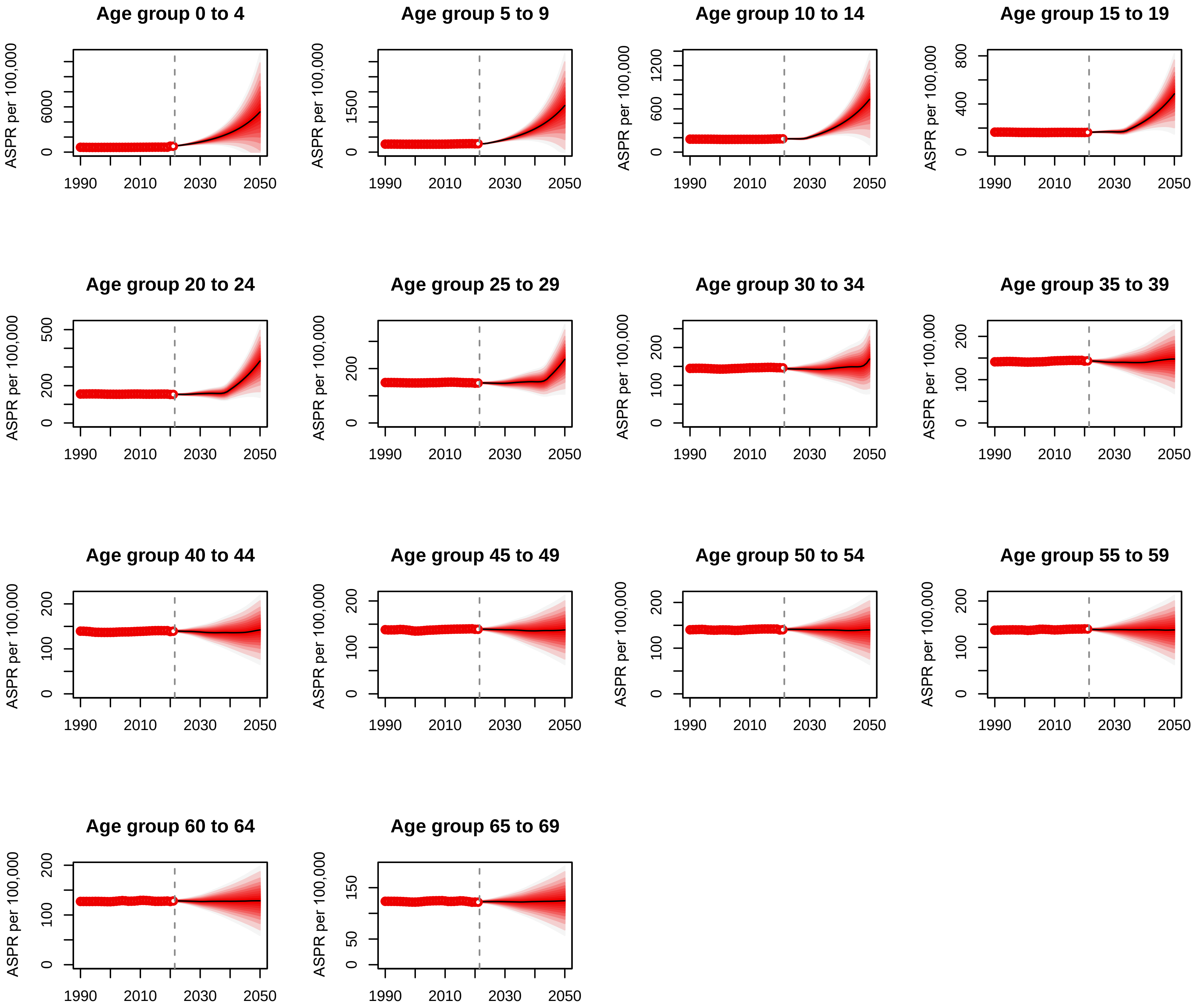
Age group 0 to 4
ASPR per 100,000
0
6000
1990
2010
2030
2050
Age group 5 to 9
ASPR per 100,000
0
1500
Age group 10 to 14
ASPR per 100,000
0
600
1200
Age group 15 to 19
ASPR per 100,000
0
400
800
Age group 20 to 24
ASPR per 100,000
0
200
500
Age group 25 to 29
ASPR per 100,000
0
200
Age group 30 to 34
ASPR per 100,000
0
100
200
Age group 35 to 39
ASPR per 100,000
0
100
200
Age group 40 to 44
ASPR per 100,000
0
100
200
Age group 45 to 49
ASPR per 100,000
0
100
200
Age group 50 to 54
ASPR per 100,000
0
100
200
Age group 55 to 59
ASPR per 100,000
0
100
200
Age group 60 to 64
ASPR per 100,000
0
100
200
Age group 65 to 69
ASPR per 100,000
0
50
150
