# Supplementary material for: Global, regional, and national burdens of congenital heart anomalies from 1990 to 2021, and projections to 2050
Source: Front Pediatr. 2025 Aug 18;13:1601620. doi: 10.3389/fped.2025.1601620 (PMC12399661; doi:10.3389/fped.2025.1601620)
Supplement: Supplementary file 12 [file Datasheet11.pdf]

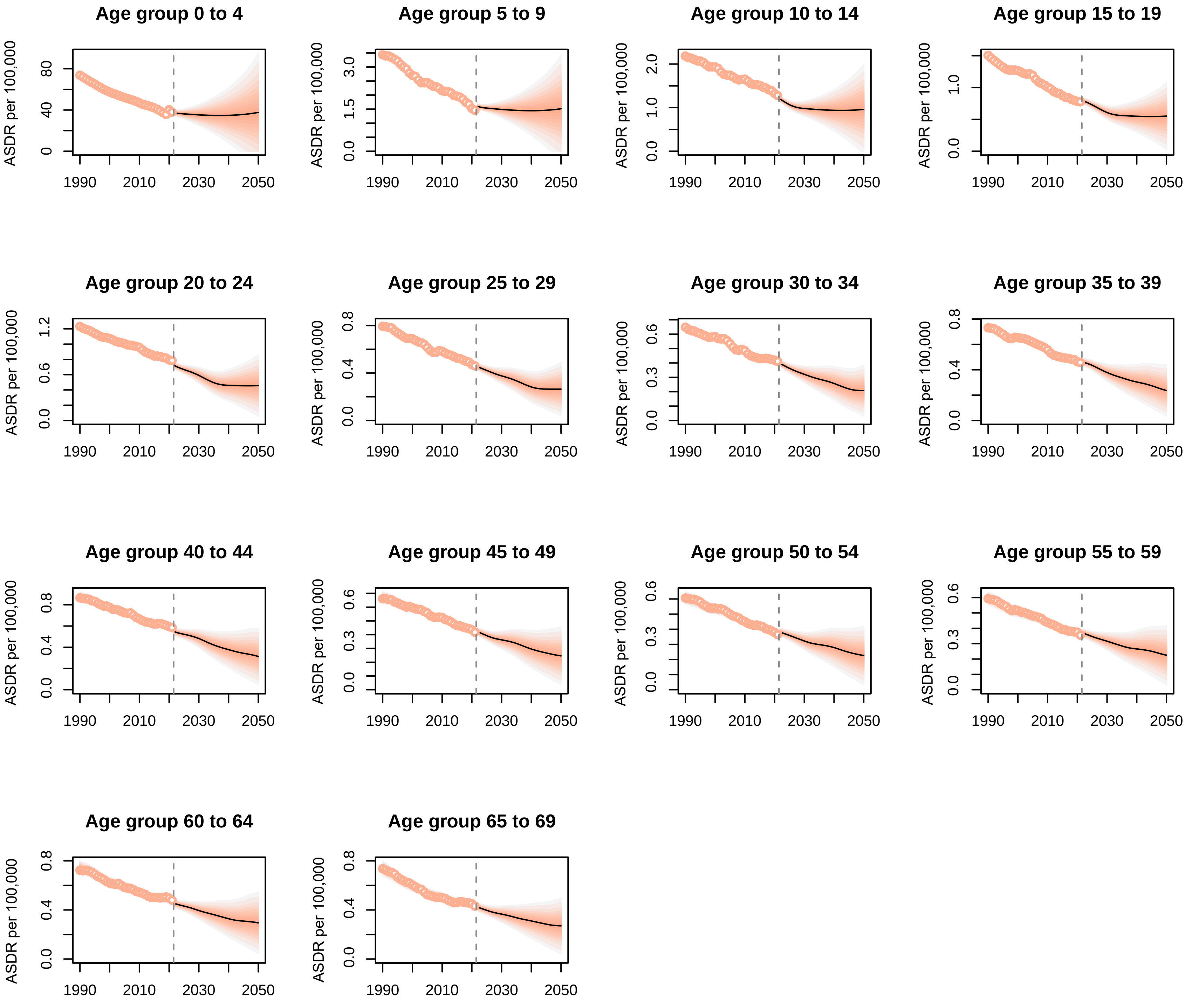

Age group 0 to 4
Age group 5 to 9
Age group 10 to 14
Age group 15 to 19
Age group 20 to 24
Age group 25 to 29
Age group 30 to 34
Age group 35 to 39
Age group 40 to 44
Age group 45 to 49
Age group 50 to 54
Age group 55 to 59
Age group 60 to 64
Age group 65 to 69
ASDR per 100,000
1990
2010
2030
2050
